# Changes in peripheral blood compounds following psychopharmacological treatment in drug-naïve first-episode patients with either schizophrenia or major depressive disorder: a meta-analysis

**DOI:** 10.1017/S0033291721000155

**Published:** 2021-03

**Authors:** Nuray Çakici, Arjen L. Sutterland, Brenda W. J. H. Penninx, Lieuwe de Haan, Nico J. M. van Beveren

**Affiliations:** 1 Department of Psychiatry and Amsterdam Neuroscience, Amsterdam UMC, University of Amsterdam, Meibergdreef 9, 1105 AZ Amsterdam, the Netherlands; 2 Parnassia Academy, Parnassia Psychiatric Institute, Kiwistraat 43, 2552 DH The Hague, the Netherlands; 3 Department of Psychiatry, Amsterdam Public Health Research Institute and Amsterdam Neuroscience, Amsterdam UMC, Vrije Universiteit, De Boelelaan 1105, 1081 HV Amsterdam, the Netherlands; 4 Department of Psychiatry, Erasmus Medical Center, Doctor Molewaterplein 40, 3015 GD Rotterdam, the Netherlands; 5 Department of Neuroscience, Erasmus Medical Center, Doctor Molewaterplein 40, 3015 GD Rotterdam, the Netherlands

**Keywords:** Cytokine, drug-naïve, first-episode, glucose metabolism, growth factors, immune system, major depressive disorder, neuroinflammation, psychopharmacological treatment, schizophrenia

## Abstract

**Background:**

This meta-analysis on peripheral blood compounds in drug-naïve first-episode patients with either schizophrenia or major depressive disorder (MDD) examined which compounds change following psychopharmacological treatment.

**Methods:**

The Embase, PubMed and PsycINFO databases were systematically searched for longitudinal studies reporting measurements of blood compounds in drug-naïve first-episode schizophrenia or MDD.

**Results:**

For this random-effects meta-analysis, we retrieved a total of 31 studies comprising 1818 schizophrenia patients, and 14 studies comprising 469 MDD patients. Brain-derived neurotrophic factor (BDNF) increased following treatment in schizophrenia (Hedges' *g* (*g*): 0.55; 95% confidence interval (CI) 0.39–0.70; *p* < 0.001) and MDD (*g*: 0.51; CI 0.06–0.96; *p* = 0.027). Interleukin (IL)-6 levels decreased in schizophrenia (*g*: −0.48; CI −0.85 to −0.11; *p* = 0.011), and for MDD a trend of decreased IL-6 levels was observed (*g*: −0.39; CI −0.87 to 0.09; *p* = 0.115). Tumor necrosis factor alpha (TNF*α*) also decreased in schizophrenia (*g*: −0.34; CI −0.68 to −0.01; *p* = 0.047) and in MDD (*g*: −1.02; CI −1.79 to −0.25; *p* = 0.009). Fasting glucose levels increased only in schizophrenia (*g*: 0.26; CI 0.07–0.44; *p* = 0.007), but not in MDD. No changes were found for C-reactive protein, IL-1*β*, IL-2 and IL-4.

**Conclusions:**

Psychopharmacological treatment has modulating effects on BDNF and TNF*α* in drug-naïve first-episode patients with either schizophrenia or MDD. These findings support efforts for further research into transdiagnostic preventive strategies and augmentation therapy for those with immune dysfunctions.

## Introduction

Historically, schizophrenia and major depressive disorder (MDD) have been regarded as separate clinical disorders, based on their distinct clinical presentation and course of the disorder. An obvious clinical argument for their distinction is the fact that both disorders are treated with pharmacologically distinct classes of therapeutics, i.e. dopamine antagonists for schizophrenia and enhancers of serotonin and/or noradrenalin activity for MDD. However, overlapping symptoms are observed such as psychotic or mood symptoms, apathy and cognitive impairment (Hill et al., 2009). Moreover, environmental and genetic risk factors are shared across both disorders (Buckholtz & Meyer-Lindenberg, 2012).

Genetic association studies have shown that patients with schizophrenia or MDD may have an immune system more prone to being activated, as expressed by major histocompatibility complex molecules (Debnath, Cannon, & Venkatasubramanian, 2013), its enhancers (Mokhtari & Lachman, 2016; Sekar et al., 2016) and complement factor 4 (Sekar et al., 2016) in schizophrenia, and associations between polymorphisms in immune genes and MDD (Barnes, Mondelli, & Pariante, 2017). Environmental stressors that activate the immune system in both disorders such as infection, trauma, stress or drug abuse, may put components of the immune system of the brain (i.e. microglia) in an altered state of activity (Barnes et al., 2017; Buckholtz & Meyer-Lindenberg, 2012; Fineberg & Ellman, 2013; Kahn et al., 2015; Lowe, Sasiadek, Coles, & George, 2019). As a result, microglia and other glia may reduce their neurotrophic function and produce less growth factors, such as brain-derived neurotrophic factor (BDNF) involved in neuroplasticity, leading to decreased neuron proliferation, resulting in reduced connectivity (Brites & Fernandes, 2015; Chew, Fusar-Poli, & Schmitz, 2013). Peripheral neurotoxic inflammatory factors may be increased by neuroinflammation (Drexhage et al., 2011; Monji, Kato, & Kanba, 2009). There is meta-analytic evidence that peripheral cytokines are altered in psychosis (Pillinger, D'Ambrosio, McCutcheon, & O, 2018a, 2018b) and in depression (Kohler et al., 2017). Meta-analytic evidence has also shown that patients with first-episode psychosis have an altered glucose homeostasis at disease onset (Greenhalgh et al., 2017; Kucukgoncu et al., 2019; Perry, McIntosh, Weich, Singh, & Rees, 2016; Pillinger et al., 2017), and that MDD increases the risks of hyperglycemia, insulin resistance and type 2 diabetes (Kan et al., 2013; Mezuk, Eaton, Albrecht, & Golden, 2008). Shared underlying factors may be responsible for the co-occurrence of immune and glucose metabolism disturbances often observed in schizophrenia and MDD (Bernardi, Marcuzzi, Piscianz, Tommasini, & Fabris, 2018; Milaneschi, Lamers, Berk, & Penninx, 2020; Nishiyama, Fujimoto, Takeuchi, & Azuma, 2018; Steiner et al., 2014).

Therefore, in recent years there has been an increasing interest in the transdiagnostic aspects of major psychiatric disorders (Van Os, Linscott, Myin-Germeys, Delespaul, & Krabbendam, 2009; Wigman et al., 2015). One of these aspects may be the effect of psychopharmacological treatment on peripheral blood compounds, as both antipsychotic and antidepressant treatment seem to have immunomodulatory properties (Capuzzi, Bartoli, Crocamo, Clerici, & Carrà, 2017; Köhler et al., 2018; Miller, Buckley, Seabolt, Mellor, & Kirkpatrick, 2011; Strawbridge et al., 2015; Wang et al., 2019). Psychopharmacological treatment also seems to have modulating effects on BDNF in schizophrenia and MDD (Fernandes et al., 2015; Sen, Duman, & Sanacora, 2008).

However, most studies report serum or plasma measurements of patients who already use psychotropic medication. Therefore, prior treatment, chronic illness or other factors associated with prolonged illness duration, such as social decline and poor lifestyle habits, could affect these measurements. Also, the similarity of altered peripheral blood compounds before and after treatment between schizophrenia and MDD has been sparsely explored. A better understanding of the underlying pathophysiology of both psychiatric disorders is mandatory in order to plan for potential broad preventive strategies and treatment regarding immune and metabolic dysfunctions prevalent in these disorders.

In the current meta-analysis, we aimed to answer the following question: which peripheral growth, immune or glucose metabolism compounds change following psychopharmacological treatment in drug-naïve first-episode patients with schizophrenia or MDD? Moreover, we aimed to answer the question whether these changes in compounds following treatment in schizophrenia or MDD are similar or dissimilar in direction and/or magnitude.

## Methods

### Literature search

**Fig. 1.**
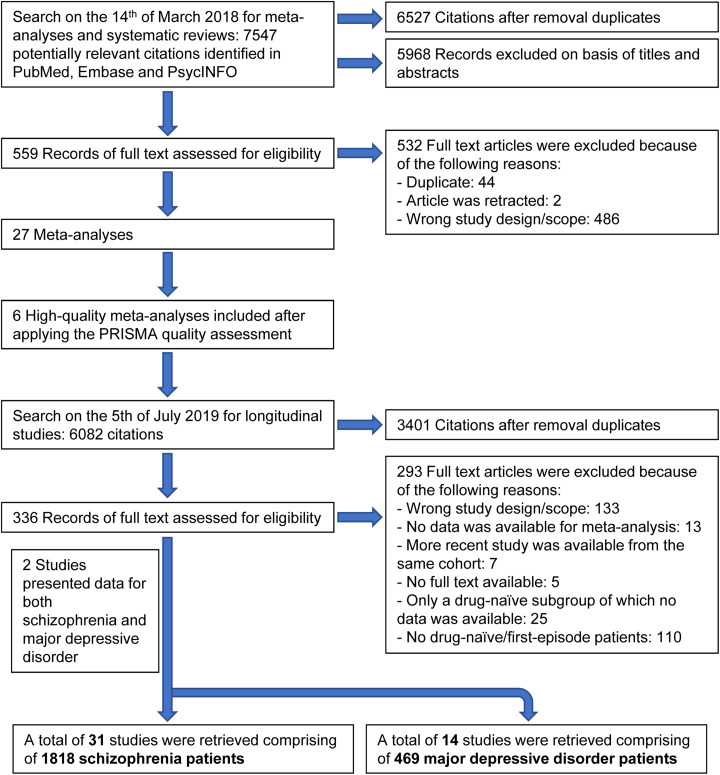
Study selection.

The literature was systematically reviewed according to the Preferred Reporting Items for Systematic Reviews and Meta-analyses and Meta-analysis of Observational Studies in Epidemiology guidelines (online Supplementary Tables S1 and S2) (Moher, Liberati, Tetzlaff, & Altman, 2009; Stroup et al., 2000). Two independent investigators (N. Ç. and N. v. B.) systematically searched the Embase, PubMed and PsycINFO databases from inception to the 5th of July 2019, without language restrictions. Details of the search strategy are provided in the online Supplementary Table S3 and Fig. 1. Finally, blood compounds were included if at least two studies were available investigating the same blood compound.

### Inclusion criteria

Inclusion criteria were (1) longitudinal studies presenting peripheral blood compound measurements before and after antipsychotic treatment in schizophrenia or antidepressant treatment in MDD; (2) a diagnosis of schizophrenia, schizophreniform disorder or MDD according to the Diagnostic and Statistical Manual of Mental Disorders or International Statistical Classification of Diseases; (3) growth and immune compounds; (4) glucose homeostasis compounds under fasting conditions; (5) antipsychotic-naïve patients with schizophrenia and antidepressant-naïve patients with MDD; (6) first-episode of schizophrenia or MDD; (7) studies reported information to calculate common effect size statistics of change scores, i.e. means and standard deviations before and after treatment; exact *p*, *t* or *z* values; or corresponding authors could supply these data upon request.

### Data extraction and processing

Two authors (N. Ç. and N. v. B.) independently extracted data from the retrieved longitudinal studies. We used data of the last observation carried forward analysis and data of completer analyses when provided. If multiple publications from the same cohort were available, we extracted data from the largest and/or most recent dataset.

In a sensitivity analysis we re-ran our analyses including only high-quality studies as assessed with the Newcastle-Ottawa quality assessment scale (NOS) (Wells et al., 2009) for which all studies should meet at least two-thirds of the NOS criteria, implying a cut-off score of 6.

### Statistical analysis

We included a blood compound for analysis if at least two studies reported measurements on the respective compound. Standardized mean differences were calculated, represented as Hedges' *g* (*g*), using a random effects model. We assessed heterogeneity across studies using the Cochran *Q* statistic (Bowden, Tierney, Copas, & Burdett, 2011). Inconsistency across studies was assessed with the *I*^2^ statistic, assigning adjectives of low, moderate and high heterogeneity to *I*^2^ values of 0 to <25%, 25–75% and >75%, respectively. (Higgins, Thompson, Deeks, & Altman, 2003). Potential publication bias was assessed using the Egger test of the intercept if 10 or more studies were analyzed for the same compound and represented diagrammatically with funnel plots (online Supplementary Fig. S1) (Egger, Davey Smith, Schneider, & Minder, 1997), and the trim and fill method (Duval & Tweedie, 2000). Meta-regression of continuous moderators was performed if at least six studies were available (Fu et al., 2011). Following this rule, we assessed the effects of age, body-mass index (BMI), illness duration, treatment duration, total baseline severity score, positive symptoms, negative symptoms [as measured with the Positive and Negative Syndrome Scale (PANSS)], Hamilton Rating Scale for Depression and sex (% males). Additionally, we compared the effect sizes statistically of each compound between schizophrenia and MDD using a Wald-type test (Pillinger et al., 2018a). Next, we statistically compared the subgroup summary effect sizes of growth, immune and glucose, by running a combined analysis of all studies assigned to the respective subgroup. Finally, we restricted our analyses to high-quality studies only to reassess the direction of change and magnitude of the compounds in schizophrenia and MDD. Results of meta-analysis and meta-regression with a *p* value <0.05 were considered significant. All analyses were performed using R software 3.4.0 and Comprehensive Meta-Analysis version 3.0 (Borenstein, Hedges, Higgins, & Rothstein, 2013; R Development Core Team, 2008).

## Results

### Retrieved studies

**Fig. 2.**
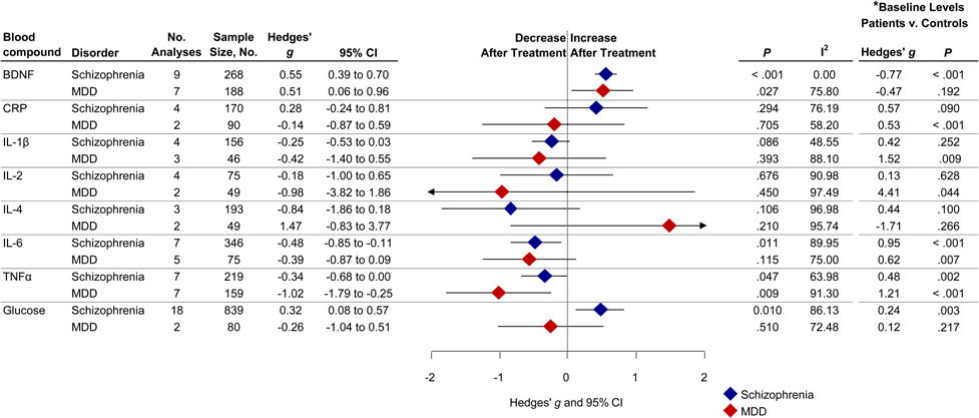
Forest plot showing effect sizes of the change of blood compounds following treatment in drug-naïve first-episode schizophrenia and MDD. Diamonds illustrate the summary effect sizes of change, the middle of each diamond represents the summary effect size, and the width of the lines depicts the width of the overall 95% CI. BDNF, brain-derived neurotrophic factor; CRP, C-reactive protein; IL, interleukin; MDD, major depressive disorder; *P*, *p* value; TNF*α*, tumor necrosis factor alpha. *Baseline levels in drug-naïve first-episode patients measured before treatment compared with healthy controls (Çakici et al., 2020).

A total of 31 studies were retrieved comprising 1818 drug-naïve first-episode schizophrenia patients, and a total of 14 studies were retrieved comprising 469 drug-naïve first-episode MDD patients. From the following eight peripheral blood compounds at least two studies were available for meta-analysis for both disorders: BDNF, C-reactive protein (CRP), interleukin (IL)-1*β*, IL-2, IL-4, IL-6, tumor necrosis factor alpha (TNF*α*) and fasting glucose concentration. The study selection process is presented in Fig. 1. The effect sizes of change for peripheral blood compounds following treatment in schizophrenia and MDD are presented in Fig. 2. Figure 2 also shows the results of (baseline) blood compounds in drug-naïve first-episode patients with schizophrenia or MDD compared with healthy controls, as published in a previous meta-analysis (Çakici et al., 2020).

Additional study information such as catchment area, patient characteristics, psychopharmacological treatment and treatment duration are provided in online Supplementary Tables S4–S6.

### Peripheral growth compounds: BDNF

**Fig. 3.**
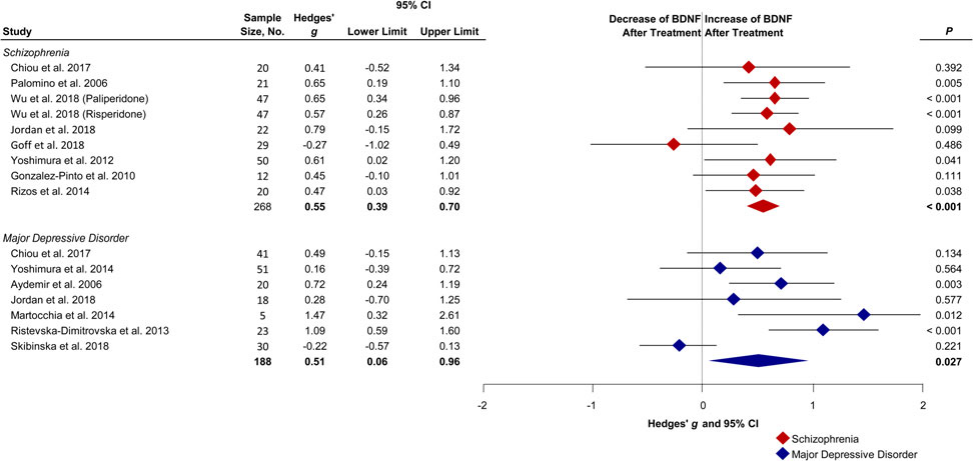
Forest plot showing effect sizes of the change of BDNF following treatment in drug-naïve first-episode schizophrenia and MDD. Baseline levels in drug-naïve first-episode schizophrenia (Hedges' *g* = −0.77; *p* < 0.001) and MDD (Hedges' *g* = −0.47; *p* = 0.192) patients measured before treatment compared with healthy controls (Çakici et al., 2020). Diamonds illustrate the summary effect sizes of change, the middle of each diamond represents the summary effect size, and the width of the lines depicts the width of the overall 95% CI. BDNF, brain-derived neurotrophic factor; *P*, *p* value.

BDNF increased in schizophrenia patients following treatment with a medium effect size (*g*: 0.55; 95% CI (CI), 0.39 to 0.70; *p* < 0.001; *I*_2_ = 0%, Fig. 3). MDD patients also showed increased BDNF following treatment with a similar effect size as compared with schizophrenia (*g*: 0.51; CI 0.06–0.96; *p* = 0.027; *I*^2^ = 76%).

### Immune compounds: CRP, cytokines and TNFα

**Fig. 4.**
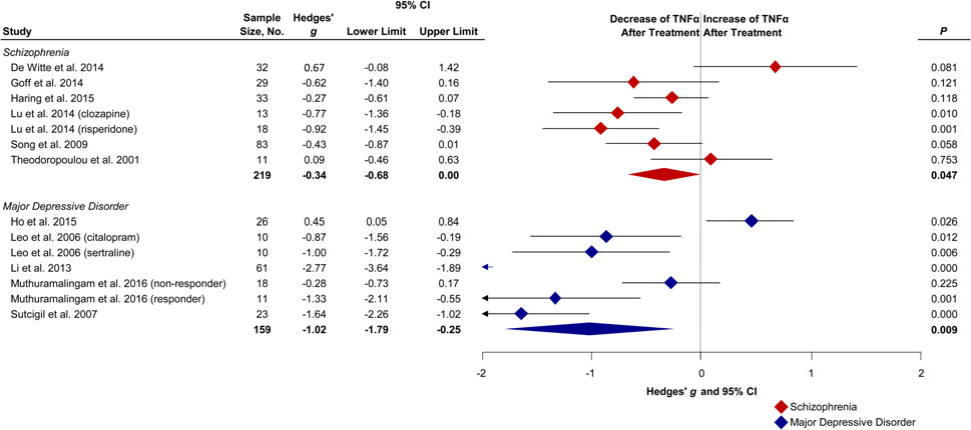
Forest plot showing effect sizes of the change of TNF*α* following treatment in drug-naïve first-episode schizophrenia and MDD. Baseline levels in drug-naïve first-episode schizophrenia (Hedges' *g* = 0.48; *p* = 0.002) and MDD (Hedges' *g* = 1.21; *p* < 0.001) patients measured before treatment compared with healthy controls (Çakici et al., 2020). Diamonds illustrate the effect sizes of change, the middle of each diamond represents the effect size, the width of the lines depicts the width of the 95% CI, and the width of the summary effect size diamond depicts the overall 95% CI. TNF*α*, tumor necrosis factor alpha; *P*, *p* value.

CRP remained unchanged following treatment in schizophrenia (*g*: 0.28; CI −0.24 to 0.81; *p* = 0.294; *I*^2^ = 76%) and in MDD (*g*: −0.14; CI −0.87 to 0.59; *p* = 0.705; *I*^2^ = 58%). IL-1*β* did not significantly change following treatment in schizophrenia (*g*: −0.25; CI −0.53 to 0.04; *p* = 0.086; *I*^2^ = 49%) and in MDD (*g*: −0.42; CI −1.40 to 0.55; *p* = 0.393; *I*^2^ = 88%). IL-2 was not significantly altered after treatment in schizophrenia patients (*g*: −0.18; CI −1.00 to 0.65; *p* = 0.676; *I*^2^ = 91%) nor after treatment in MDD patients (*g*: −0.98; CI −3.82 to 1.86; *p* = 0.50; *I*^2^ = 97%). IL-4 decreased non-significantly following treatment in schizophrenia (*g*: −0.84; CI −1.86 to 0.18; *p* = 0.11; *I*^2^ = 97%) whereas in MDD IL-4 increased non-significantly (*g*: 1.47; CI −0.83 to 3.78; *p* = 0.210; *I*^2^ = 96%). The Wald type test did not find a significant difference between groups (*p* = 0.070). IL-6 decreased in schizophrenia patients following treatment with a medium effect size (*g*: −0.48; CI −0.85 to −0.11; *p* = 0.011; *I*^2^ = 90%). In MDD we observed a trend of decreased IL-6 levels with a small to medium effect size (*g*: −0.39; CI −0.87 to 0.09; *p* = 0.115; *I*^2^ = 75%). TNF*α* decreased following treatment in schizophrenia with a small to medium effect size (*g*: −0.34; CI −0.68 to −0.01; *p* = 0.047; *I*^2^ = 64%, Fig. 4). MDD patients also showed decreased TNF*α* after treatment with a large effect size (*g*: −1.02; CI −1.79 to −0.25; *p* = 0.009; *I*^2^ = 91%) compared with schizophrenia patients. See online Supplementary Figs S2–S6 for effect size estimates for individual studies.

### Glucose metabolism compounds: glucose

Glucose increased in schizophrenia patients following antipsychotic treatment with a small to medium effect size (*g*: 0.32; CI 0.08–0.57; *p* = 0.010; *I*^2^ = 86%). Indication of publication bias was found (Egger test: *p* < 0.001). After correcting for publication bias glucose remained increased following treatment in schizophrenia (*g*: 0.32; CI 0.08–0.57). Glucose remained unchanged in MDD patients following treatment (*g*: −0.26; CI −1.04 to 0.52; *p* = 0.51; *I*^2^ = 72%). See online Supplementary Fig. S7 for effect size estimates for individual studies.

### Effects of moderators

Meta-regression analysis showed that schizophrenia patients with a high symptom severity score before treatment had a greater change of IL-6 levels following treatment (*g*: −0.03; CI −0.04 to −0.01; *p* = 0.002) (Table 1, online Supplementary Fig. S8). This effect was greatly influenced by the negative symptom score before treatment, i.e. patients with more negative symptoms before treatment showed a greater change in IL-6 levels after treatment (*g*: −0.13; −0.21 to −0.06; *p* < 0.001; online Supplementary Fig. S9). Younger patients with schizophrenia showed more change in TNF*α* levels following treatment compared with older patients with schizophrenia (*g*: 0.14; CI 0.02–0.26; *p* = 0.019; online Supplementary Fig. S10). A longer treatment duration was associated with a greater change in TNF*α* levels after treatment in MDD patients (*g*: −0.64; CI −0.91 to −0.37; *p* < 0.001; online Supplementary Fig. S11). Schizophrenia patients with lower BMI before treatment showed a greater change in glucose levels following treatment (*g*: −0.53; CI −0.87 to −0.18; *p* = 0.003; online Supplementary Fig. S12). Male schizophrenia patients tended to have a smaller change in TNF*α* after treatment compared with female schizophrenia patients (*g*: 0.08; CI 0.03–0.12; *p* < 0.001; online Supplementary Fig. S12). Besides these effects, meta-regression analysis showed no significant effects for illness duration, change of BMI and change of symptom severity on the changes in blood compounds levels following treatment.

**Table 1.** Effects of moderators

Schizophrenia						Major Depressive Disorder					
Blood Compound	No. Analyses	Coefficient	95% Lower Limit	95% Upper Limit	*P*	Blood compound	No. Analyses	Coefficient	95% Lower Limit	95% Upper Limit	*P*
***BDNF***						***BDNF***					
Age	8	0.04	-0.04	0.11	0.341	Age	7	0.03	-0.01	0.07	0.135
Sex (% males)	6	0.00	-0.01	0.02	0.660	Sex (% males)	7	0.01	−0.01	0.02	0.537
Treatment duration	9	0.00	−0.01	0.01	0.824	Treatment duration	7	0.08	−0.22	0.38	0.606
***CRP***	n/a					***CRP***	n/a				
***Glucose***						***Glucose***	n/a				
Age	17	0.01	−0.09	0.10	0.912						
Treatment duration	17	−0.04	−0.12	0.05	0.401	***IL-1β***	n/a				
BMI, before treatment	9	−0.53	−0.87	−0.18	0.003						
BMI, change score	6	0.04	−0.53	0.61	0.886	***IL-4***	n/a				
Illness duration	6	−0.22	−1.44	0.99	0.720						
PANSS total, pre-treatment	8	−0.01	−0.03	0.02	0.693	***IL-6***	n/a				
PANSS total, change score	6	0.01	−0.02	0.03	0.703						
PANSS positive, pre-treatment	7	0.02	−0.07	0.10	0.740	***TNFα***					
PANSS positive, change score	6	0.02	−0.05	0.09	0.623	Treatment duration	7	−0.64	−0.91	−0.37	< 0.001
PANSS negative, pre-treatment	7	−0.03	−0.14	0.09	0.651						
PANSS negative, change score	6	0.02	−0.09	0.13	0.729						
Sex (% males)	13	−0.01	−0.03	0.01	0.253						
***IL-1β***	n/a										
***IL-4***	n/a										
***IL-6***											
Age	7	−0.06	−0.15	0.04	0.243						
Treatment duration	7	0.02	−0.02	0.05	0.360						
PANSS total, pre-treatment	7	−0.03	−0.04	−0.01	0.002						
PANSS total, change score	6	−0.01	−0.04	0.02	0.449						
PANSS positive, pre-treatment	7	−0.10	−0.20	0.01	0.061						
PANSS negative, pre-treatment	7	−0.13	−0.21	−0.06	0.001						
PANSS negative, change score	6	−0.05	−0.14	0.04	0.291						
Sex (% males)	7	0.04	−0.03	0.10	0.302						
***TNFα***											
Age	7	0.14	0.02	0.26	0.019						
Sex (% males)	6	0.08	0.03	0.12	< 0.001						
Treatment duration	7	0.00	−0.05	0.04	0.930						

BMI, body-mass index; BDNF, brain-derived neurotrophic factor; CRP, C-reactive protein; IL, interleukin; n/a, not enough studies available for analysis; PANSS, Positive and Negative Syndrome Scale; TNF*α*, tumor necrosis factor alpha.

Meta-regression of continuous moderators was performed if at least six studies were available.

### Statistical comparison of effect sizes

For IL-4 we observed a non-significant difference of the overall effect size in schizophrenia *v.* MDD (Wald score: −1.80; *p* = 0.070; online Supplementary Table S7). For all other compounds there was no significant difference between the overall effect size of alterations in schizophrenia *v.* MDD. There was no significant difference between the overall effect size of alterations of immune compounds in schizophrenia *v.* MDD.

### Effect of study quality

In a sensitivity analysis including only high-quality studies (online Supplementary Tables S8 and S9), our results of increased BDNF after treatment in schizophrenia remained the same with a similar effect size. Similar to the main analysis, we observed decreased TNF*α* in MDD after treatment, but with a larger effect size (*g*: −2.25; *p* = 0.045). In contrast to the initial findings, we did not observe any changes for IL-6, TNF*α* and glucose in schizophrenia, nor for BDNF and IL-6 in MDD. However, due to the relatively small number of available studies for this sensitivity analysis we probably had insufficient power (as evidenced by the generally smaller effect sizes) to make conclusive statements.

## Discussion

In this meta-analysis, we found that drug-naïve first-episode patients with either schizophrenia or MDD both show changes of peripheral blood compounds in a similar direction following treatment: *growth factor* BDNF increased with a similar medium effect size and *immune factor* TNF*α* decreased in schizophrenia with a small to medium effect size and in MDD with a large effect size. *Glucose metabolism factor* fasting glucose increased in schizophrenia patients after treatment with a small to medium effect size, but not in MDD patients. We found no significant differences among the overall effect sizes of all compounds between both disorders.

Below we will discuss findings concerning growth, immune and glucose metabolism factors separately in more detail.

### Impaired neuroplasticity

Impairment of neuroplasticity has been implicated in the pathophysiology of both schizophrenia and MDD (Rao et al., 2017). BDNF, among other neurotrophic factors, contribute to maintaining the plasticity of neurons and is therefore a key component for cognition (Benraiss, Chmielnicki, Lerner, Roh, & Goldman, 2001; Pencea, Bingaman, Wiegand, & Luskin, 2001). There is meta-analytic evidence that BDNF is decreased in both schizophrenia and MDD present from disease-onset (Çakici et al., 2020; Fernandes et al., 2015; Molendijk et al., 2014).

In the current study, BDNF significantly increased following treatment in both disorders. These findings suggest that treating a current first-episode of schizophrenia or MDD using regular psychopharmacological treatment tends to restore BDNF levels. It is unclear what the exact nature of this restoration is. We could not discern whether increased BDNF levels are caused by psychopharmacological treatment or by other factors associated with reduction of symptom severity. Rat studies have shown that second-generation antipsychotics (SGAs) have neuroprotective effects through modulation of BDNF levels (Angelucci, Mathe, & Aloe, 2000; Bai, Chlan-Fourney, Bowen, Keegan, & Li, 2003; Wakade, Mahadik, Waller, & Chiu, 2002), and that antidepressants increase total BDNF messenger RNA expression in astrocytes and microglia (Hisaoka-Nakashima et al., 2016).

Mounting evidence shows that hippocampal atrophy is associated with MDD (Duman & Monteggia, 2006; Schmidt & Duman, 2007), already from disease-onset (Cole, Costafreda, McGuffin, & Fu, 2011) and that antidepressants can block or even reverse hippocampal atrophy (Schmidt & Duman, 2007). It is thought that the neurotrophic actions of antidepressants upregulate BDNF which, in turn, increases neurogenesis within the hippocampus (Schmidt & Duman, 2007). Additionally, electroconvulsive therapy can increase brain volume in the limbic structures, including the hippocampus and amygdala (Takamiya et al., 2018), and increase BDNF among patients with MDD (Birkenhager, Geldermans, Van den Broek, van Beveren, & Fekkes, 2012; Rocha et al., 2016).

According to a meta-analysis of longitudinal studies, BDNF might be regarded as a biomarker for successful treatment of MDD (Polyakova et al., 2015). Polyakova and colleagues showed that BDNF was initially decreased in acute MDD and subsequently increased following antidepressant treatment in patients that in particular responded to treatment (Polyakova et al., 2015). A meta-analysis of drug-free psychosis patients showed that BDNF increased after antipsychotic treatment in psychosis patients, however treatment response was not related to the increase of BDNF over the course of treatment (Fernandes et al., 2015).

### Immune system dysfunctions, partly modifiable?

A growing body of evidence shows that signs of low-grade peripheral inflammation are seen in both schizophrenia and MDD from disease-onset (Çakici et al., 2020; Goldsmith, Rapaport, & Miller, 2016; Kohler et al., 2017; Upthegrove, Manzanares-Teson, & Barnes, 2014). In a previous meta-analysis we showed that increased immune factors IL-6 and TNF*α* are present in drug-naïve first-episode patients with schizophrenia or MDD compared with healthy controls (Çakici et al., 2020). In the current study, we found that TNF*α* substantially decreased following treatment in both drug-naïve first-episode patient groups. IL-6 decreased following treatment in schizophrenia patients and for MDD patients we observed a trend of decreased IL-6 following treatment. Two studies which were not included in the main analysis investigated the influence of non-pharmacological treatment on IL-6 in drug-naïve first-episode patients with MDD (Gazal et al., 2013; Keri, Szabo, & Kelemen, 2014), using cognitive-behavioral therapy (CBT), a widely used evidence-based treatment for MDD (American Psychiatric Association, 2000). Interestingly, when adding these CBT studies in a post-hoc analysis to the main IL-6 meta-analysis (Gazal et al., 2013; Keri et al., 2014), we observed a significant decrease of IL-6 following treatment in MDD patients (online Supplementary Table S6B). These CBT findings may suggest that the modulating effects on IL-6 and possibly other immune markers are not limited to solely treatment with medication (Keri et al., 2014). Indeed, other factors associated with reduction of symptom severity, such as improvement in sleep and diet, or diminished stress, may also have beneficial anti-inflammatory effects.

Both *in vitro* and *in vivo* research has shown that particularly atypical antipsychotics possess anti-inflammatory properties. Clozapine displays anti-inflammatory and neuroprotective effects (Hu et al., 2012), but also olanzapine, aripiprazole and risperidone have shown to possess anti-inflammatory effects (Juncal-Ruiz et al., 2018; Obuchowicz, Bielecka-Wajdman, Paul-Samojedny, & Nowacka, 2017; Stapel et al., 2018). In MDD, antidepressants have proven to counteract inflammation and hypothalamic–pituitary–adrenal axis activation (Eskeland, Halvorsen, & Tanum, 2017; Roumestan et al., 2007). However, it was not possible in our meta-analysis to differentiate between the anti-inflammatory actions across the different types of psychopharmacological treatments, since SGA and selective serotonin reuptake inhibitor (SSRI) studies are overrepresented in the literature and other agents are underrepresented (online Supplementary Table S10).

We like to point out that the effect sizes of change point consistently into the direction of normalization. In other words, when the direction of effect size of a compound was increased or decreased at baseline, the direction of effect size of this compound after treatment seemed to decrease or increase respectively. This was most notable with BDNF, TNF*α* and IL-6. Although not significant, IL-4 seemed to be altered in opposite directions before treatment in both disorders, and subsequently changed in opposite directions after treatment in both disorders. These observations should be cautiously interpreted due to the paucity of available data and subsequent lack of power in this meta-analysis, but merit further investigation.

In a similar meta-analysis, IL-6 and IL-1*β* decreased after 4 weeks of antipsychotic treatment (Capuzzi et al., 2017). Furthermore, they presented decreased IL-2 and no change of TNF*α* after treatment. These differences may be explained because we included more studies for each compound and extracted data from the largest or most recent dataset. Similar to our findings, another meta-analysis reported increased IL-6, decreased IL-1*β* and no change of IL-2 following treatment in acute psychosis, and decreased IL-6 and unaltered IL-2 following treatment in acute depression (Goldsmith et al., 2016). In contrast to our study, no change in TNF*α* was reported in both disorders and an increase of IL-1*β* was observed in acute depression. These differences could have been influenced by effects of medication or prolonged disease duration, as the majority of the included studies was not performed in drug-naïve subjects, and not all patients were in their first-episode of disease (Goldsmith et al., 2016). A more recent meta-analysis investigated the effects of antidepressant treatment on cytokines and chemokines in MDD patients – of whom the majority were not drug-naïve or in their first-episode (Köhler et al., 2018). Similar to our study, they presented decreased IL-6 and TNF*α*, and no changes for IL-1*β*, IL-2 and IL-4 following treatment in MDD (Köhler et al., 2018).

Taken together, both immune factors IL-6 and TNF*α* are increased in schizophrenia and MDD at disease onset. Treatment seems to normalize IL-6 and TNF*α* in both disorders.

### Inflammatory subtypes

Signs of low-grade inflammation could indicate that immune alterations are disease-inherent abnormalities, however immune alterations could also be present only in some patients. A meta-analysis of immune parameters observed lower variability of especially IL-6 in psychosis patients, possibly indicating that immune alterations in schizophrenia patients are truly a sign of intrinsic immune dysfunction (Pillinger et al., 2018a, 2018b). Conversely, another study found genetic evidence for an immune-related subgroup of schizophrenia (Trossbach et al., 2019).

MDD is a highly heterogeneous disorder and the presence of an inflammatory MDD subtype could be relevant. Lamers and colleagues presented evidence that patients with specific atypical features show associations with immuno-metabolic outcomes including IL-6, CRP and metabolic syndrome components, both cross-sectionally and longitudinally (Lamers et al., 2020). Hence, they proposed an immunometabolic depression model – including chronic low-grade inflammation, oxidative stress, disruption of neuroendocrine regulators (leptin and insulin resistance) and biomolecules (dyslipidemia) involved in energy metabolism – predominantly present in patients with atypical behavioral symptoms which could explain the considerable comorbidity between depression and cardiometabolic conditions (e.g. obesity, metabolic syndrome and diabetes) (Milaneschi et al., 2020). However, a meta-analysis looking at variability of immune parameters in untreated depression, found that depression is associated with a pro-inflammatory state, rather than with an immune subgroup, as some of the inflammatory markers elevated in depression, including CRP and IL-12, showed low variability (Osimo et al., 2020).

High inflammation before the start of treatment in both depression and schizophrenia seems to be associated with worse treatment response. A multinational, multi-center, randomized, double-blind study of first-episode psychosis patients showed that those with the most severe symptoms before treatment presented with higher pro-inflammatory compounds and were the most at risk of non-remission compared to psychosis patients with less severe symptoms (Martinuzzi et al., 2019). Additionally, in a longitudinal study of first-episode psychosis patients an association was found between increased high-sensitivity CRP, triglycerides and BMI at baseline, and higher PANSS scores and reduced treatment response at 1-year follow-up (Nettis et al., 2019).

In depression, regular antidepressant treatment fails for over 30% of the patients, whereby those with high inflammation and/or high BMI are in particular at risk for resistance to treatment (Bekhbat et al., 2018; Haroon et al., 2018). A longitudinal study showed that high IL-6 at baseline could predict a chronic course of depression (Lamers et al., 2019). Moreover, a meta-analysis showed that increased inflammation is contributory to treatment resistance in depression (Strawbridge et al., 2015).

In our meta-regression analyses, the changes in blood compounds were merely related to symptom severity. The presence of inflammatory subtypes could be an explanatory factor, however, to evaluate predictors of response we would need individual patient data, which is beyond the scope of the current study.

### Glucose homeostasis

In this meta-analysis, we observed an increase of fasting glucose levels following treatment in drug-naïve first-episode schizophrenia patients, but not in those with MDD. Meta-analytic evidence has confirmed an altered glucose homeostasis is already present in schizophrenia from disease onset (Çakici et al., 2020; Greenhalgh et al., 2017; Kucukgoncu et al., 2019; Perry et al., 2016; Pillinger et al., 2017), even in drug-naïve patients (Çakici et al., 2020). Indeed, long before the advent of antipsychotics an increased incidence of diabetes among schizophrenia patients has been described (Kohen, 2004). SGAs, especially olanzapine and clozapine, have well-known side-effects such as obesity and metabolic syndrome, in particular abnormal glucose and lipid metabolism (Pramyothin & Khaodhiar, 2010). Given the evidence that an altered glucose homeostasis is already present at disease onset in schizophrenia (Çakici et al., 2020; Pillinger et al., 2017), antipsychotic treatment could be a contributory factor to further compromising the glucose homeostasis, resulting in diabetes or other metabolic diseases, often observed in chronic schizophrenia.

In contrast, an altered glucose homeostasis does not seem to be present in first-onset MDD, and, neither does treatment in first-episode MDD contribute to glucose homeostasis alteration. It should be noted that for the current meta-analysis only two studies were available that investigated the effects of antidepressant treatment on glucose changes in MDD patients. Additionally, metabolic dysregulation or disorders are often seen in patients who have been affected by MDD for a longer time (Lasserre et al., 2017) Additionally, metabolic diseases are often seen in patients who have been affected by MDD for a longer time (Lasserre et al., 2017). A bidirectional relationship might exist between obesity-related traits and MDD with atypical features, as a large international consortium study showed that patients with atypical depression carried a higher number of genetic risk variants for disturbances in BMI, CRP and leptin (Milaneschi et al., 2017). Also, a cross-disorder proteomics analysis, using the same multi-analyte platform, showed that increased levels of insulin and leptin are present in both schizophrenia and MDD (Çakici et al., 2019a).

Taken together, evidence was found for a dysfunctional glucose metabolism in drug-naïve first-episode schizophrenia patients before and after psychotropic treatment, but not in those with depression. However, impaired glucose homeostasis and other metabolic alterations are often observed in patients with chronic MDD. Diabetes and cardiovascular diseases are more common in schizophrenia and MDD compared to the healthy population, and contribute to a decreased lifespan in patients with schizophrenia or MDD. It is unclear whether this association is simply reflective of side effects of psychotropic medication or an unhealthy lifestyle, or whether glucose and metabolic alterations are also the results of specific disease-inherent abnormalities.

### Clinical implications: paving the way for personal diagnostics and targeted treatment

Given the possibility of common underlying pathways for immune dysfunction and impaired plasticity between schizophrenia and MDD, it is worthwhile to consider the clinical relevance of our findings for treatment. The presence of inflammation before the start of treatment in both depression and schizophrenia seems to be associated with worse treatment response, as outlined in the section ‘Inflammatory subtypes’. Signs of inflammation in schizophrenia and MDD readily suggest augmentation therapy for those patients in which the underlying pathophysiology is related to inflammatory dysfunctions. Although antipsychotics already have some anti-inflammatory actions (Hu et al., 2012), a meta-analysis on the efficacy of augmentation with anti-inflammatory agents for schizophrenia patients showed that some anti-inflammatory agents improved symptomatology, i.e. aspirin, estrogens, minocycline and *N*-acetylcysteine (Çakici, van Beveren, Judge-Hundal, Koola, & Sommer, 2019b). TNF antagonism seems to be particularly effective in patients with treatment-resistant depression, signs of inflammation (CRP > 5 mg/L) and elevated plasma lipids and cholesterols (Bekhbat et al., 2018). Furthermore, there is meta-analytic evidence that anti-inflammatory add-on treatment to antidepressants may have beneficial effects on improving symptom severity in MDD (Köhler-Forsberg et al., 2019).

Taken together, our findings support the hypothesis that factors associated with neuroplasticity and inflammation are dysregulated in the early-phase of both schizophrenia and MDD. Although regular treatment appears to normalize some growth and immune factors, anti-inflammatory augmentation therapy seems beneficial in schizophrenia and MDD, especially given that an inflammatory subtype is associated with worse treatment response. Further research is warranted to identify which biological underlying disease mechanisms are responsible. The latter readily suggests research into advanced diagnostics and augmentation therapy for inflammatory or metabolic dysfunctions present in schizophrenia and MDD. Further research may elucidate a serum-based clinical decision support system in schizophrenia and MDD to identify inflammatory and/or metabolic aberrant subgroups, in order to augment their regular therapy with anti-inflammatory or metabolic medications.

### Limitations

To our best knowledge, this is the first meta-analysis evaluating changes in peripheral growth, immune and glucose metabolism compounds following psychopharmacological treatment in drug-naïve first-episode patients with schizophrenia or MDD. Based on our approach to include only drug-naïve first-episode patients, we could make a better estimation of the alterations in compounds following treatment measured in peripheral blood, without the influence of prior treatment, chronic illness or other factors associated with prolonged illness duration, such as social decline, poorer dietary habits and insufficient physical activity. Ideally, for a meta-analysis, a sufficient number of studies and sample sizes is needed (e.g. at least five studies). For BDNF and TNF*α* there was a sufficient number of studies available for analysis. The other peripheral blood compounds, that were analyzed in less than five studies (such as IL-6), merit further investigation. Unfortunately, evidence regarding the change of blood compounds following treatment in drug-naïve first-episode patients with schizophrenia or MDD is relatively scarce. Therefore, we could not include other peripheral blood compounds that are often associated with schizophrenia (e.g. nerve growth factor (NGF), IL-8 and insulin) (Çakici et al., 2020; Upthegrove et al., 2014) or MDD (e.g. soluble IL-2 receptor, NGF and vascular endothelial growth factor) (Çakici et al., 2020; Kohler et al., 2017). Furthermore, since SGA and SSRI studies are overrepresented in the literature, we could not differentiate between the anti-inflammatory actions across the different types of psychopharmacological treatments (online Supplementary Table S10).

Another important limitation is that we did not include other major psychiatric disorders such as bipolar disorder. Generally, schizophrenia and bipolar disorder are seen as more related to one another than schizophrenia and MDD. As a consequence, there is a substantial body of research investigating similarities between schizophrenia and bipolar disorder, both from a clinical perspective, as well as the biological underpinnings of both disorders. Our study focused on schizophrenia and MDD, but future projects are warranted to compare bipolar disorder with these disorders. Current findings point at transdiagnostic biological disturbances, underscoring the importance of studying neurobiologic disturbance more broadly in psychiatric disorders.

## Conclusions

In this meta-analysis, we found evidence that growth factor BDNF increase and immune factor TNF*α* decrease following treatment in drug-naïve first-episode patients with either schizophrenia or MDD. BDNF and TNF*α* are possibly shared markers of an active first-episode of schizophrenia and MDD, since these blood compounds tend to normalize following treatment. An altered glucose homeostasis might be uniquely present at the onset of schizophrenia, with further dysregulation by antipsychotic treatment. Our findings support efforts for further research into transdiagnostic preventive strategies and augmentation therapy for those with high inflammation or metabolic disturbances. Currently, it may help patients with schizophrenia or MDD to accept and continue regular (psychopharmacological) treatment when they are told that dysregulated growth and immune factors tend to normalize during treatment.

## Supplementary material

For supplementary material accompanying this paper visit https://doi.org/10.1017/S0033291721000155. click here to view supplementary material
