# Supplementary material for: Changes in peripheral blood compounds following psychopharmacological treatment in drug-naïve first-episode patients with either schizophrenia or major depressive disorder: a meta-analysis
Source: Psychol Med. 2021 Mar 3;51(4):538–49. doi: 10.1017/S0033291721000155 (PMC8020491; doi:10.1017/S0033291721000155)
Supplement: Supplementary file 1 [file S0033291721000155sup001.docx]

**Supplementary Material**

**Index**

**Table 1.** Preferred Reporting Items for Systematic Reviews and Meta-analyses *p.* 2

(PRISMA)

**Table 2.** Meta-analyses Of Observational Studies in Epidemiology (MOOSE) Checklist *p.* 4

**Figure 1.** Funnel Plot of Glucose Analysis in Schizophrenia *p. 6*

**Table 3.** Search Strategy *p. 7*

**Table 4.** Studies Investigating the Change of Peripheral Blood Compounds *p. 11*

Following Treatment in Drug-Naïve First-Episode Patients With Schizophrenia

or Major Depressive Disorder

**Table 5.** Additional Study Characteristics *p.*17

**Table 6.** Inclusion and Exclusion Criteria Used by the Included Studies *p.* 19

**Table 7.** Statistical Comparison of Effect Sizes in Schizophrenia versus MDD Using a *p. 24*

Wald-Type Test

**Table 8.** Newcastle-Ottawa Quality Assessment Scale for Cohort Studies *p.* 25

**Table 9.** Effect Sizes for Compounds Measured in High-Quality Studies Only  *p.* 27

**Table 10.** Effects of Psychopharmacological Treatment Types on Effect Sizes *p*. 28

**Figure 2.** C-Reactive Protein (CRP) *p.* 30

**Figure 3.** Interleukin (IL)-1β *p.* 31

**Figure 4.** IL-2 *p.* 32

**Figure 5.** IL-4 *p*. 33

**Figure 6A.** IL-6 *p.* 34

**Figure 6B.** IL-6 (including two psychotherapy studies) *p.* 35

**Figure 7.** Fasting Glucose Concentration *p.* 36

**Figure 8.** Regression of Hedges' *g* on PANSS Total (Pre-Treatment) and Change of *p.* 37

IL-6 After Treatment

**Figure 9.** Regression of Hedges' *g* on PANSS Negative (Pre-Treatment) and Change *p.* 38

of IL-6 After Treatment

**Figure 10.** Regression of Hedges' *g* on Age and Change of TNFα After Treatment *p.* 39

**Figure 11.** Regression of Hedges' *g* on Treatment Duration in MDD and Change of *p.* 40

TNFα After Treatment

**Figure 12.** Regression of Hedges’ *g* on body-mass index (BMI) (Pre-Treatment) and *p.* 41

Change of Glucose After Treatment

**Figure 13.** Regression of Hedges’ *g* on Sex (% Males) and Change of TNFα after *p*. 42

Treatment

**References**  *p.* 43

| **Table 1. PRISMA (Preferred Reporting Items for Systematic Reviews and Meta-analyses)** | | | | | | | | |
| --- | --- | --- | --- | --- | --- | --- | --- | --- |
|  |  |  |  |  |  |  |  |  |
| **Section/topic** | | **#** | | **Checklist item** | | **Reported on page #** | | |
| **TITLE** | |  | |  | |  | | |
| Title | | 1 | | Identify the report as a systematic review, meta-analysis, or both. | | 1 | | |
| **ABSTRACT** | |  | |  | |  | | |
| Structured summary | | 2 | | Provide a structured summary including, as applicable: background; objectives; data sources; study eligibility criteria, participants, and interventions; study appraisal and synthesis methods; results; limitations; conclusions and implications of key findings; systematic review registration number. | | 3 | | |
| **INTRODUCTION** | |  | |  | |  | | |
| Rationale | | 3 | | Describe the rationale for the review in the context of what is already known. | | 5 | | |
| Objectives | | 4 | | Provide an explicit statement of questions being addressed with reference to participants, interventions, comparisons, outcomes, and study design (PICOS). | | 5 | | |
| **METHODS** | |  | |  | |  | | |
| Protocol and registration | | 5 | | Indicate if a review protocol exists, if and where it can be accessed (e.g., Web address), and, if available, provide registration information including registration number. | | <https://www.crd.york.ac.uk/>  prospero/display_record.  php?RecordID=102039 PROSPERO 2018 CRD42018102039 | | |
| Eligibility criteria | | 6 | | Specify study characteristics (e.g., PICOS, length of follow-up) and report characteristics (e.g., years considered, language, publication status) used as criteria for eligibility, giving rationale. | | 8 | | |
| Information sources | | 7 | | Describe all information sources (e.g., databases with dates of coverage, contact with study authors to identify additional studies) in the search and date last searched. | | 8 | | |
| Search | | 8 | | Present full electronic search strategy for at least one database, including any limits used, such that it could be repeated. | | Supplementary material; pages 6,7,10 | | |
| Study selection | | 9 | | State the process for selecting studies (i.e., screening, eligibility, included in systematic review, and, if applicable, included in the meta-analysis). | | 8,9; Supplementary material pages 6 and 7 | | |
| Data collection process | | 10 | | Describe method of data extraction from reports (e.g., piloted forms, independently, in duplicate) and any processes for obtaining and confirming data from investigators. | | 9 | | |
| Data items | | 11 | | List and define all variables for which data were sought (e.g., PICOS, funding sources) and any assumptions and simplifications made. | | 9 | | |
| Risk of bias in individual studies | | 12 | | Describe methods used for assessing risk of bias of individual studies | | 10; Supplementary table 9 and 10 | | |
| **Table 1 (continued). PRISMA (Preferred Reporting Items for Systematic Reviews and Meta-analyses)** | | | | | | | | |
|  |  |  |  |  |  |  |  |  |
| **Section/topic** | **#** | | **Checklist item** | | | | | **Reported on page #** |
|  |  | | information is to be used in any data synthesis. | | | | |  |
| Summary measures | 13 | | State the principal summary measures (e.g., risk ratio, difference in means). | | | | | 10 |
| Synthesis of results | 14 | | Describe the methods of handling data and combining results of studies, if done, including measures of consistency (e.g., I2) for each meta-analysis. | | | | | 10 |
| Risk of bias across studies | 15 | | Specify any assessment of risk of bias that may affect the cumulative evidence (e.g., publication bias, selective reporting within studies). | | | | | 10 |
| Additional analyses | 16 | | Describe methods of additional analyses (e.g., sensitivity or subgroup analyses, meta-regression), if done, indicating which were pre-specified. | | | | | 10 |
| **RESULTS** |  | |  | | | | |  |
| Study selection | 17 | | Give numbers of studies screened, assessed for eligibility, and included in the review, with reasons for exclusions at each stage, ideally with a flow diagram. | | | | | 13  Supplementary Figure 2 |
| Study characteristics | 18 | | For each study, present characteristics for which data were extracted (e.g., study size, PICOS, follow-up period) and provide the citations. 7 | | | | | 13  Supplementary tables 4-6 |
| Risk of bias within studies | 19 | | Present data on risk of bias of each study and, if available, any outcome level assessment (see item 12). | | | | | 14  Supplementary tables 9 and 10 |
| Results of individual studies | 20 | | For all outcomes considered (benefits or harms), present, for each study: (a) simple summary data for each intervention group (b) effect estimates and confidence intervals, ideally with a forest plot. | | | | | 13-15  Supplementary Figures 3-10 |
| Synthesis of results | 21 | | Present results of each meta-analysis done, including confidence intervals and measures of consistency. | | | | | 13-15 Supplementary Figures 3-10 |
| Risk of bias across studies | 22 | | Present results of any assessment of risk of bias across studies (see Item 15). | | | | | 16  Supplementary Tables 9 and 10 |
| Additional analysis | 23 | | Give results of additional analyses, if done (e.g., sensitivity or subgroup analyses, meta-regression [see Item 16]). | | | | | 15 Supplementary table 7 |
| **DISCUSSION** |  | |  | | | | |  |
| Summary of evidence | 24 | | Summarize the main findings including the strength of evidence for each main outcome; consider their relevance to key groups (e.g., healthcare providers, users, and policy makers). | | | | | 17, 25-27 |
| Limitations | 25 | | Discuss limitations at study and outcome level (e.g., risk of bias), and at review-level (e.g., incomplete retrieval of identified research, reporting bias). | | | | | 28,29 |
| Conclusions | 26 | | Provide a general interpretation of the results in the context of other evidence, and implications for future research. | | | | | 30 |
| **FUNDING** |  | |  | | | | |  |
| Funding | 27 | | Describe sources of funding for the systematic review and other support (e.g., supply of data); role of funders for the systematic review. | | | | | 31 |
| **Table 2. (Meta-analyses Of Observational Studies in Epidemiology) Checklist** | | | | | | | | |
|  |  |  |  |  |  |  |  |  |
| **Reporting Criteria** | | | | | **Reported (Yes/No)** | | **Reported on Page No.** | |
| **Reporting of Background** | | | | |  | |  | |
| Problem definition | | | | | Yes | | 5-7 | |
| Hypothesis statement | | | | | Yes | | 5-7 | |
| Description of Study Outcome(s) | | | | | Yes | | 5-7 | |
| Type of exposure or intervention used | | | | | Yes | | 5-7 | |
| Type of study design used | | | | | Yes | | 5-7 | |
| Study population | | | | | Yes | | 5-7 | |
| **Reporting of Search Strategy** | | | | |  | |  | |
| Qualifications of searchers (eg, librarians and investigators) | | | | | Yes | | 8 | |
| Search strategy, including time period included in the synthesis and keywords | | | | | Yes | | 8 | |
| Effort to include all available studies, including contact with authors | | | | | Yes | | 9 | |
| Databases and registries searched | | | | | Yes | | 8 | |
| Search software used, name and version, including special features used (eg, explosion) | | | | | Yes | | 8 | |
| Use of hand searching (eg, reference lists of obtained articles) | | | | | Yes | | 9 | |
| List of citations located and those excluded, including justification | | | | | Yes | | Supplementary Material Table 3 and Supplementary references | |
| Method for addressing articles published in languages other than English | | | | | Yes | | 8 | |
| Method of handling abstracts and unpublished studies | | | | | Yes | | 9 | |
| Description of any contact with authors | | | | | Yes | | 9 | |
| **Reporting of Methods** | | | | |  | |  | |
| Description of relevance or appropriateness of studies assembled for assessing the hypothesis to be tested | | | | | Yes | | 8,9 | |
| Rationale for the selection and coding of data (eg, sound clinical principles or convenience) | | | | | Yes | | 9-10 | |
| Documentation of how data were classified and coded (eg, multiple raters, blinding, and interrater reliability) | | | | | Yes | | 10 | |
| Assessment of confounding (eg, comparability of cases and controls in studies where appropriate | | | | | Yes | | 10 | |
| **Reporting Criteria** | | | | |  | |  | |
| Assessment of study quality, including blinding of quality assessors; stratification or regression on possible predictors of study results | | | | | Yes | | 10 | |

| **Table 2 (continued). (Meta-analyses Of Observational Studies in Epidemiology) Checklist** | | |
| --- | --- | --- |
|  |  |  |
| Description of statistical methods (eg, complete description of fixed or random effects models, justification of whether the chosen models account for predictors of study results, dose-response models, or cumulative meta-analysis) in sufficient detail to be replicated | Yes | 10-11 |
| Provision of appropriate tables and graphics | Yes | 13-16 |
| **Reporting of Results** |  |  |
| Table giving descriptive information for each study included | Yes | Supplementary tables 4-6 |
| Results of sensitivity testing (eg, subgroup analysis) | Yes | 15,16 Supplementary tables 7 and 10 |
| Indication of statistical uncertainty of findings | Yes | 13-15 |
| **Reporting of Discussion** |  |  |
| Quantitative assessment of bias (eg, publication bias) | Yes | 16 |
| Justification for exclusion (eg, exclusion of non–English-language citations) | Yes | 28,29 |
| Assessment of quality of included studies | Yes | 16 |
| **Reporting of Conclusions** |  |  |
| Consideration of alternative explanations for observed results |  | 25 |
| Generalization of the conclusions (ie, appropriate for the data presented and within the domain of the literature review) | Yes | 30 |
| Guidelines for future research | Yes | 30 |
| Disclosure of funding source | Yes | 31 |

**Figure 1. Funnel Plot of Glucose Analysis in Schizophrenia**

**
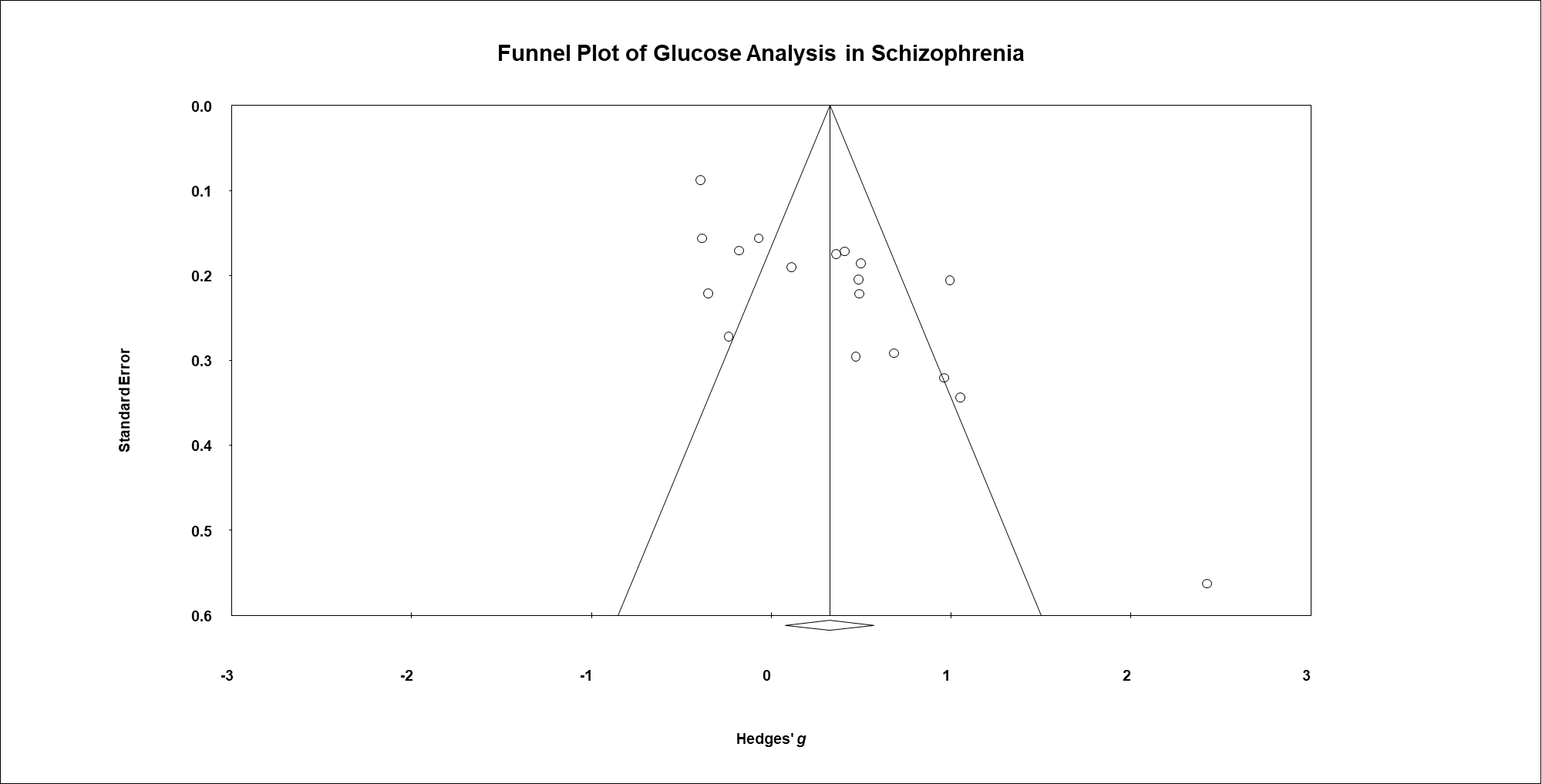
**

**Table 3. Search Strategy**

The literature was systematically reviewed according to PRISMA (Preferred Reporting Items for Systematic Reviews and Meta-analyses) and MOOSE (Meta-analysis of Observational Studies in Epidemiology) guidelines (Supplementary Table 1 and 2). Two independent investigators (N.Ç. and N.v.B.) searched the Embase, PubMed and PsycINFO databases from inception to the July 5th, 2019, for meta-analyses, systematic reviews and longitudinal studies that reported measurements of growth, immune and glucose metabolism factors measured in peripheral blood of drug-naïve first-episode patients with either schizophrenia or MDD. We first screened for meta-analyses and systematic reviews that systematically searched for peripheral blood compounds in schizophrenia and MDD, which did not necessarily restrict their inclusions to drug-naïve or first-episode patients. We only extracted longitudinal studies from meta-analyses or systematic reviews with high quality. An overview of the included meta-analyses and the AMSTAR checklist can be found on pages 8 and 9. A high quality means that the systematic review or meta-analysis provides an accurate and comprehensive summary of the results of the available studies that address the research question. For our final inclusion of studies, we screened for drug-naïve first-episode longitudinal studies starting from the last search date of inclusion in the retrieved meta-analyses or systematic reviews. If high-quality meta-analyses or systematic reviews were not available for a respective compound, we searched for drug-naïve first-episode longitudinal studies that reported measurements of these compounds from inception. No language restrictions were applied. Authors were contacted if additional data was needed for analysis (e.g., if drug-naïve or first-episode patients were only a subset of the total study population). We contacted authors for full report of relevant unpublished studies.

We completed the search by hand-searching additional relevant meta-analyses, systematic reviews and case-control studies. Disagreements were resolved by discussion with another author (L.d.H.).

| **Overview of Meta-Analyses Used for Screening for Eligible Case-Control Studies** | | | | | | |
| --- | --- | --- | --- | --- | --- | --- |
| **Analyte** | **Most Recent Meta-Analysis Used [Schizophrenia]** | **Most Recent Meta-Analysis Used [Major Depressive Disorder]** | **Databases Used By Meta-Analysis** | **Date Last Search in Meta-Analysis** | **PRISMA Quality Assessment** | **Dates searched for case-control studies** |
| BDNF | Fernandes et al, 2015 |  | Medline, Embase, the Cochrane Library, Scielo, PsycInfo, Scopus and Web  of Knowledge | March, 2014 | High | January 1st 2014 - January 19th, 2019 |
| BDNF |  | Molendijk et al, 2014 | PubMed, Embase and PsychInfo | April 1st, 2013 | High | January 1st 2013 - January 19th, 2019 |
| Cytokines (e.g., IL-1β, IL-1-RA, sIL-2r, IL-2, IL-3, IL-4, IL-6, IL-8, IL-12, IL-17, IL-18, IL-27, IFN, IFN-γ, NGF, TGF-β, TNF-α) | Upthegrove et al, 2014 |  | PubMed, EMBASE, PsycINFO, and Cochrane | June 17th, 2013 | High | January 1st 2013 - January 19th, 2019 |
| Cytokines (e.g., CCL2, CCL3, IL-1b, IL-1Ra, IL-2, sIL-2R, IL-4, IL-5, IL-6, sIL-6R, IL-8, IL-10, IL-12, IL-13, IL-17, IL-18 , CCL-3, IL-17, TNF-alpha, sTNFR-2) |  | Köhler et al, 2017 | PubMed/  MEDLINE, EMBASE, and PsycINFO | May 30th, 2016 | High | January 1st 2016 - January 19th, 2019 |
| Glucose Metabolism Compounds | Pillinger et al, 2017 |  | MEDLINE (from 1946 to week 2 of April 2016),  EMBASE (from 1947 to April 25, 2016), and PsycINFO (from  1806 to week 2 of April 2016). | MEDLINE (from 1946 to week 2 of April 2016),  EMBASE (from 1947 to April 25, 2016), and PsycINFO (from  1806 to week 2 of April 2016). | High | January 1st 2016 - January 19th, 2019 |
| Glucose Metabolism Compounds |  | Not available |  |  |  | Inception - January 19th, 2019 |
| NGF | Rao et al, 2017 |  |  | May 1st, 2016 | High | January 1st 2016 - January 19th, 2019 |
| NGF |  | Rao et al, 2017 |  | May 1st, 2016 | High | January 1st 2016 - January 19th, 2019 |
| Leptin | Not available | Not available |  |  |  | Inception - January 19th, 2019 |
| CRP, EGF, VEGF, FGF, C4 | Not available | Not available |  |  |  | Inception - January 19th, 2019 |

| **AMSTAR Checklist** | **Fernandes et al, 2015** | **Molendijk et al, 2014** | **Upthegrove et al, 2014** | **Köhler et al, 2017** | **Pillinger et al, 2017** | **Rao et al, 2017** |
| --- | --- | --- | --- | --- | --- | --- |
| 1. Did the research questions and inclusion criteria for the review include the components of PICO? | Yes | Yes | Yes | Yes | Yes | Yes |
| 2. Did the report of the review contain an explicit statement that the review methods were established prior to the conduct of the review and did the report justify any significant deviations from the protocol? | Yes | Yes | Yes | Yes | Yes | Yes |
| 3. Did the review authors explain their selection of the study designs for inclusion in the review? | Yes | Yes | Yes | Yes | Yes | Yes |
| 4. Did the review authors use a comprehensive literature search strategy? | Yes | Yes | Yes | Yes | Yes | Yes |
| 5. Did the review authors perform study selection in duplicate? | Yes | Yes | Yes | Yes | Yes | Yes |
| 6. Did the review authors perform data extraction in duplicate? | No | Yes | Yes | Yes | Yes | Yes |
| 7. Did the review authors provide a list of excluded studies and justify the exclusions? | Yes | Yes | Yes | Yes | Yes | Yes |
| 8. Did the review authors describe the included studies in adequate detail? | Yes | Yes | Yes | Yes | Yes | Yes |
| 9. Did the review authors use a satisfactory technique for assessing the risk of bias (RoB) in individual studies that were included in the review? | Yes | No | No | No | Yes | Yes |
| 10. Did the review authors report on the sources of funding for the studies included in the review? | Yes | Yes | Yes | Yes | Yes | Yes |
| 11. If meta-analysis was performed did the review authors use appropriate methods for statistical combination of results? RCTs | Yes | Yes | No | Yes | Yes | Yes |
| 12. If meta-analysis was performed, did the review authors assess the potential impact of RoB in individual studies on the results of the meta-analysis or other evidence synthesis? | Yes | Yes | Yes | Yes | Yes | Yes |
| 13. Did the review authors account for RoB in individual studies when interpreting/ discussing the results of the review? | Yes | Yes | Yes | Yes | Yes | Yes |
| 14. Did the review authors provide a satisfactory explanation for, and discussion of, any heterogeneity observed in the results of the review? | Yes | Yes | Yes | Yes | Yes | Yes |
| 15. If they performed quantitative synthesis did the review authors carry out an adequate investigation of publication bias (small study bias) and discuss its likely impact on the results of the review? | Yes | Yes | Yes | Yes | Yes | Yes |
| 16. Did the review authors report any potential sources of conflict of interest, including any funding they received for conducting the review? | Yes | Yes | Yes | Yes | Yes | Yes |
|  |  |  |  |  |  |  |
| **Quality Rating** | **High** | **High** | **High** | **High** | **High** | **High** |
|  |  |  |  |  |  |  |
|  |  |  |  |  |  |  |
|  |  |  |  |  |  |  |
|  |  |  |  |  |  |  |

**Search Terms Used to Search for Systematic Reviews and Meta-Analyses**

"Schizophrenia Spectrum and Other Psychotic Disorders" OR "Psychotic Disorders" OR "Schizophrenia" or schizophren* or psychos* or psychot* or "Depression" OR "Depressive Disorder" OR "Depressive Disorder, Major" or depression or depressive or unipolar OR affective

Immune System" OR "Complement System Proteins" OR "Lymphocytes" OR "Antibodies" OR "Biomarkers" OR "Oxidative Stress" OR "Cytokines" OR "Proteomics" OR "Inflammation" OR "Neuroimmunomodulation" OR "Hormones" OR "Serum" OR "Plasma" OR "Blood" OR "Diabetes Mellitus" OR "Glucose Intolerance" OR "Insulin Resistance" OR "Glucose Tolerance Test" or antioxidants or zinc or immune system or protein or proteins or lymphocyte* or antibod* or autoantibod* or biomarker* or oxida* or zinc or cytokine* or antioxid* or proteomics or inflammat* or neuroimmun* or hormone* or cortisol or cortisone or hydrocortisone or dexamethasone or serum or plasma or blood or diabet* or prediabet* or glucose or insulin or "Leptin" or leptin or interleukin* or tnf a or tnf alpha or tnfa or tumor necrosis factor or lymphocyte* or bdnf or brain derived neurotrophic factor or crp

Systematic OR systematic review OR meta-analysis

**Search Terms Used to Search for Longitudinal Studies**

"Schizophrenia Spectrum and Other Psychotic Disorders" OR "Psychotic Disorders" OR "Schizophrenia" OR schizophren* OR psychosis OR psychot*

“Depression" OR "Depressive Disorder" OR "Depressive Disorder, Major" OR depression OR depressive OR unipolar OR affective

"Diabetes Mellitus" OR "Glucose Intolerance" OR "Insulin Resistance" OR "Glucose Tolerance Test" OR "Hyperglycemia" OR diabet* OR prediabet* OR glucose OR insulin OR hyperglycemia OR impaired fasting glucose OR impaired glucose tolerance OR impaired plasma glucose OR OGTT OR homeosta* OR hba1c OR IGF* OR "Adiponectin" OR adiponectin* OR "C-Peptide" OR C-peptide OR "Insulin" OR "Insulin-Secreting Cells"

"Cytokines" OR cytokin* OR immune* OR inflammat* OR pro-inflammat* OR anti-inflammat* OR "Interleukins" OR interleukin* OR "Tumor Necrosis Factor-alpha" OR Tumor Necrosis Factor-alpha OR TNF-alpha OR IFN* OR TGF*

"Cytokines" OR cytokin* OR immune* OR inflammat* OR pro-inflammat* OR anti-inflammat* OR "Interleukins" OR interleukin* OR "Tumor Necrosis Factor-alpha" OR Tumor Necrosis Factor-alpha OR TNF-alpha

"Brain-Derived Neurotrophic Factor" OR Brain-Derived Neurotrophic Factor OR BDNF

"Nerve Growth Factor" OR Nerve Growth Factor OR NGF

"Leptin" OR leptin OR "C-Reactive Protein" OR C-Reactive Protein OR CRP OR growth factor OR "Complement C4" OR Complement C4 OR soluble TNF receptor* OR Soluble Tumor Necrosis Factor

first episode* OR FEP OR early onset OR at risk OR ultra high risk OR prodrome OR naïve OR drug naïve OR medication naïve OR antipsychotic naïve OR neuroleptic naïve OR unmedicated OR nonmedicated OR non-medicated OR untreated OR newly diagnosed OR never treated

| **Table 4. Studies Investigating the Change of** **Peripheral Blood Compounds Following Treatment in Drug-Naïve First-Episode Patients With Schizophrenia or Major Depressive Disorder** | | | | | | | | | | |
| --- | --- | --- | --- | --- | --- | --- | --- | --- | --- | --- |
| **Study** | **Sample size** | **Male/female** | **Age, mean (SD)** | **BMI** | **Disorder** | **Illness duration, mean (SD)** | **Compounds** | **Treatment type and dose** | **Follow-up period** | **Baseline total symptom severity, mean (SD)** |
| Aydemir et al, 2006 (Turkey) | 20 | 0/20 | 35.55 (7.58) |  | MDD |  | BDNF | Aripiprazole and risperidone (n=13); haloperidol and olanzapine groups (n=12) | 6 weeks | HDRS: 39.75 (7.40) |
| Basoglu et al, 2010 (Turkey) | 20 | 20/0 | 21.2 (0.75) | 22.0 (2.2) | Schizophrenia |  | FG | Olanzapine 20 mg (starting dose: 10 mg) | 2 and 6 weeks | PANSS:  95.2 (14.8) |
| Borovcanin et al, 2013 (Serbia) | 88 | 36/52 | 33.64 (8.84) |  | Schizophrenia | 0.28 (1.93) years | IL-4, IL-6 | Atypical antipsychotics (n=26); typical antipsychotics (n=14) | 30 days | PANSS: 100.96 (14.76) |
| Cai et al, 2012 (China) | 11 | 6/5 | 27.6 (9.5) | 21.0 (1.7) | Schizophrenia | 0.8 (0.6) years | FG |  | 3 and 6 weeks | PANSS: 55.6 (16.8) |
| Chang et al, 2013 (Taiwan) | 50 |  | 38.6 (11.2) |  | MDD |  | FG | Fluoxetine (n=28, 24.0 ± 8.2 mg); venlafaxine (n=22 77.8 ± 10.8 mg) | 6 weeks |  |
| Chang et al, 2017 (Taiwan) | 72 | 21/51 | 40.72 (12.45) |  | MDD |  | CRP | Fluoxetine 20-80 mg or venlafaxine 37.5-225 mg | 6 weeks | HDRS: 9.82 (7.57) |
| Chiou et al, 2017 (Taiwan) | 34 | 12/22 | 30.6 (11.2) | 22.7 (4.5) | Schizophrenia |  | BDNF | Risperidone (1–6 mg, mean dose 3.8mg daily), n=16; olanzapine (10mg daily), n=1; Zotepine (200mg daily), n=1; paliperidone (mean dose 6mg daily) n=2 | 4 weeks | PANSS: 121.3 (13.7) |
| Chiou et al, 2017 (Taiwan) | 71 | 15/56 | 37.4 (10.5) | 21.9 (3.8) | MDD |  |  | Escitalopram (dose range: 10-20 mg/d), fluoxetine (dose range: 20-40 mg/d), mirtazapine (dose range: 30–60 mg/d), paroxetine (dose range: 20–40 mg/d), or venlafaxine (dose range: 75–225 mg/d) | 26 days | 17 item HDRM: 34.5 (4.4) |
| De Witte et al, 2014 (Germany/the Netherlands) | 32 | 22/10 | 31.2 (10.5) | 24.0 (4.4) | Schizophrenia |  | TNFα | Olanzapine, quetiapine, risperidone, or mix of different antipsychotics | 6 weeks | PANSS: 21.5 (6.5) |
| Ding et al, 2014 (China) | 69 | 37/32 | 27.48 (7.75) | 20.19 (1.98) | Schizophrenia | 9.68 (9.06) months | IL-6 | Risperidone 2 to 6 mg | 4 weeks | PANSS: 77.72 (11.02) |
| Goff et al, 2018 (China) | 29 |  | 25.2 |  | Schizophrenia or schizophreniform disorder |  | BDNF, CRP, IL-1β, TNF-α |  | 8 months |  |
| Gonzalez-Pinto et al, 2010 (Spain) | 12 |  | 22.46 (5.84) |  | Schizophrenia |  | BDNF | Olanzapine 5-20 mg | 1, 6 and 12 months |  |
| Haring et al, 2015 (Estonia) | 33 | 19/14 | 25.8 (5.8) | 22.8 (2.8) | Schizophrenia and schizophreniform disorder | < 3 years | IL-1β, IL-2, IL-4, IL-6, TNF-α | Atypical (n = 25), typical (n = 1) or mixed (n=6) antipsychotic medication | 7 months | PANSS:  111.44 (20.67) |
| Ho et al, 2015 (Taiwan) | 26 | NA | 23.2 (3.2) |  | MDD (n=14), dysthymic disorder (n=10), and depressive disorder not otherwise specified (n=2) | 12.9 (17.4) months | IL-10, IL-1β, IL-2, IL-4, IL-6, TNF-α | [Escitalopram](mailto:Escitalopram@) | 4 weeks | HDRS: 18.5 (6.7) |
| Huang et al, 2018 (China) | 29 | 20/9 | 23.79 (5.89) | 23.0 (3.0) | Schizophrenia |  | FG | Olanzapine 5 mg/d | 13 weeks | PANSS: 88.44 (13.55) |
| Huang et al, 2018 (China) | 28 | 17/11 | 21.54 (5.60) |  | Schizophrenia |  | FG | Paliperidone palmitate injection | 13 weeks | PANSS: 87.39 (20.46) |
| Jordan et al, 2018 (Germany) | 22 | 13/9 | *30.00 (25.00, 35.50) | *22.14 (20.99, 26.00) | Schizophrenia |  | BDNF | Olanzapine (n = 7), quetiapine (n = 7) and risperidone (n = 8) | 6 weeks | PANSS:  *****88.0 (73.8, 101.5) |
| Jordan et al, 2018 (Germany) | 18 | 11/7 | *46.00 (33.50, 52.75) | *24.51 (21.46, 26.36) | MDD |  | BDNF | Mirtazapine (n = 10) and venlafaxine (n = 8) | 6 weeks | *****HAMD-21: 21.0 (15.8, 24.3) |
| Kavzoglu and Hariri et al, 2013 (Turkey) | 50 | 25/25 | 30.14 (7.50) |  | Schizophrenia |  | FG | Risperidone (n=13), olanzapine (n=12), clozapine (n=3), amisulpride (n=1), quetiapine (n=1), haloperidol (n=2), combination of typical and atypical antipsychotics (n= 2), and a combination of two typical antipsychotics (n=5) | 3 months |  |
| Leo et al, 2006 (Italy) | 46 | 20/26 | 34.85 (5.88) |  | MDD |  | IL-1β, IL-6, TNF-α, FG | Sertraline (n=10, 100 mg); citalopram (n=10, 20 mg) | 6 weeks | HAM-D: 22.63 (5.14) |
| Li et al, 2013 (China) | 61 | 11/50 | 31.77 (6.50) | 21.54 (2.07) | MDD | 3.08 (1.06) months | TNF-α | Venlafaxine 75 mg daily for 5 to 7 days and gradually increasing to 150—225 mg daily. | 8 weeks | HRSD-17: 22.8 (2.0) |
| Lin et al, 2018 (China) | 12 | 10/2 83.333 | 27.08 (10.18) | 21.97 (2.77) | Schizophrenia | 9.92 (7.86) months | IL-1β, TNF-α | Olanzapine | 4 and 8 weeks | PANSS:  79.50 (14.70) |
| Lu et al, 2004 (China) | 31 | 16/15 | 23.18 (5.44) |  | Schizophrenia | 5.89 (6.54) months | IL-2, IL-6, TNF-α | Clozapine or risperidone | 6 months | PANSS:  89.62 (14.35) |
| Martocchia et al, 2014 (Italy) | 5 | 2/3 | 74.0 (6.8) | < 30 | MDD |  | BDNF | Escitalopram 10 mg | 2 months |  |
| Muthuramalingam et al, 2016 (India) | 29 | 9/20 | 35.5 (8.9) |  | MDD |  | IL-6, TNF-α | Fluoxetine | 6 weeks |  |
| Narula et al, 2010 (India) | 34 | 22/12 | 31 (10.09) | 20.2 (3.92) | Schizophrenia |  | FG | Olanzapine (5-20 mg/d) | 12 weeks | PANSS:  103.82 (12.7) |
| Narula et al, 2010 (India) | 33 | 22/11 | 31.21 (9.70) | 20.56 (3.85) | Schizophrenia |  | FG | Olanzapine (5-20 mg/d) and topiramate (50 mg and after first week increased to 100 mg/d) | 12 weeks | PANSS:  102.85 (17.5) |
| Noto et al, 2016 (Brazil) | 72 | 42/30 | 25.77 (7.92) |  | Schizophrenia and schizophreniform disorder |  | IL-4, IL-6, TNF-α | Risperidone, mean dose of 4.0 mg/day (SD=2.1) | 11 weeks | PANSS:  96.1 (21.4) |
| Palomino et al, 2006 (Spain) | 21 |  | 23.7 (S.E.M.: ±11) |  | Schizophrenia |  | BDNF | Atypical antipsychotics (62–68%), with lithium or other mood stabilizers together with atypical antipsychotics (23–26%), with typical antipsychotics (7–11%), or received no treatment (2–4%)^#^ | 1, 6 and 12 months |  |
| Ristevska-Dimitrovska et al, 2013 [Macedonian study] | 23 | 12/11 | 44.22 [20-72] |  | MDD |  | BDNF | Sertraline, paroxetine or venlafaxine | 8 weeks | HDRS: 28.52 (4.02) |
| Rizos et al, 2014  (Greece) | 14 | 8/6 | 29.71 (10.21) |  | Schizophrenia | 8.00 (4.60) months | BDNF | SGAs: 4 patients were treated with olanzapine, 4 with quetiapine, 2 with risperidone, 2 with ripiprazole and 2 with amisulpride. | 8 months |  |
| Saddichha et al, 2008 (India) | 99 | 52/47 | 26.0 (5.5) |  | Schizophrenia | 7 months | FG | Olanzapine (n=35, 16.5 ± 4.6 mg); risperidone (n=33, 4.4 ± 1.2 mg) and haloperidol (n=31, 13.4 ± 3.6 mg) | 6 weeks |  |
| Skibinska et al, 2018 (Poland) | 30 | 0/30 | 38.06 (10.21) |  | MDD |  | BDNF, FG | Sertraline 50 mg or venlafaxine 75 mg (Three patients had their dose of sertraline increased to 100 mg/day. Two patients receiving venlafaxine had their doses increased to 150 and 225 mg/day) | 8 weeks | HDRS-17: 23.9 (4.27) |
| Song et al, 2009 (China) | 83 | 43/40 | 27.3 (6.7) |  | Schizophrenia | 7 months | IL-1β, TNF-α | Fluoxetine 20 mg~~ | 4 weeks | PANSS: 89.1 (11.6) |
| Song et al, 2014 (China) | 62 | 33/29 | 24.7 (5.5) | 20.14 (2.02) | Schizophrenia | 7.1 (5.7) months | BDNF, IL-6 | Risperidone 2-6 mg (benzodiazepines only for insomnia and anticholinergic agents for dystonia reaction) | 2 weeks, 1, 2, 3 and 6 months | PANSS: 71.8 (8.0) |
| Stefanović et al, 2015 (Serbia) | 78 | 33/45 |  |  | Schizophrenia |  | CRP | First-generation antipsychotics (N=38); second generation antipsychotics (N=22) and combined antipsychotic therapy (N=18). Haloperidol (2-15 mg): ±12.7 mg at admission and ± 8.8 mg at dismissal. Risperidone (2-6 mg): ±3.6 mg. Olanzapine (2-20 mg): ±8.3 mg. Clozapine (25-125 mg): ±67.3. | 30 days | PANSS: 100.1 (13.0) |
| Sutcigil et al, 2007 (Turkey) | 23 |  | 34.78 (7.42) |  | MDD |  | IL-2, IL-4, TNF-α | Sertraline (50-100 mg) | 8 weeks | HDRS: 28.39 (4.53) |
| Theodoropoulou et al, 2001 (Greece) | 53 | 33/20 | 24 [16–41] |  | Schizophrenia |  | IL-1β, IL-2, TNF-α | Neuroleptic treatment |  |  |
| Wani et al, 2015 (India) | 50 | 32/18 | 25.40 (4.85) |  | Schizophrenia |  | FG |  | 6 and 14 weeks |  |
| Wu et al, 2018 (China) | 94 | 69/25 | 28.95 (4.85) |  | Schizophrenia | **1.5 [0.5-2.7] years | BDNF | Risperidone or Paliperidone treatment | 12 weeks | PANSS: 81.02 (19.43) |
| Yoshimura et al, 2012 (Japan) | 50 | 29/21 | 30.8 (5.3) |  | Schizophrenia |  | BDNF | Aripiprazole (6-30 mg) | 8 weeks | PANSS: 75.0 (8.1) |
| Yoshimura et al, 2014 (Japan) | 51 | 19/32 | 38 (19) |  | MDD | 2.1 (0.9) months |  | Fluvoxamine | 4 weeks | HAMD: 19.3 (2.8) |
| Yuan et al, 2018 (China) | 41 | 23/18 | 23.1 (8.0) | 20.54 (2.52) | Schizophrenia | 5.9 (3.0) months | CRP, FG | Risperidone (start 1 mg/d to 4–6 mg/d) | 24 weeks | PANSS: 82.3 (12.7) |
| Zhai et al, 2017 (China) | 407 |  | 25.3 (8.8) | 21.3 (3.4) | Schizophrenia | 5.1 (6.5) months | FG | Atypical antipsychotic treatment, N=381; Atypical antipsychotic treatment, N=175; Combined therapy polypharmacy, N=228; Antipsychotic comedication, N=161; Antidepressant, N=35; Lithium, N=99.^#^ | 1-8 weeks |  |
| Zhou et al, 2019 (China) | 22 | 11/11 | 24.18 (7.04) | 22.0 (4.75) | Schizophrenia | 20.52 (19.66) months | FG | Aripiprazole (mean: 20.0 mg/d) | 12 weeks | PANSS:  99.95 (10.04) |
| Zhou et al, 2019 (China) | 21 | 11/10 | 28.24 (10.57) | 21.11 (2.77) | Schizophrenia | 14.63 (21.01) | FG | Olanzapine (mean: 15.4 mg/d) | 12 weeks | PANSS:  104.29 (25.83) |
| Abbreviations: BDNF, brain-derived neurotrophic factor; CRP, C-reactive protein; d, day; FG, fasting glucose; HDRM, Hamilton Depression Rating Scale; HAM-D, Hamilton Rating Scale for Depression; IL, interleukin; Major Depressive Disorder; PANSS, Positive and Negative Syndrome Scale; TNFα, tumor necrosis factor alpha. ***** = median, quartile 1 and quartile 3. | | | | | | | | | | |

| **Table 5. Additional study characteristics** | | | | | | |
| --- | --- | --- | --- | --- | --- | --- |
| **Study** | **Setting (area)** | **Allowance of other neuroleptic use at baseline, e.g. benzodiazepines** | **Sample source** | **Fasting state** | **Assay** | **Diagnostic criteria (Structured interview)** |
| Aydemir et al, 2006 | Turkey (Ankara) | No | Serum | NA | ELISA | DSM-IV (SCID) |
| Basoglu et al, 2010 | Turkey (Ankara) | No | Plasma | Yes | NA | DSM-IV |
| Borovcanin et al, 2013 | Serbia (Belgrade) | No | Serum | NA | ELISA | ICD-10 |
| Cai et al, 2012 | China (Changsha) | Alprazolam and benzhexol | Plasma | Yes | Varian INOVA600MHz spectrometer | DSM (SCID-I) |
| Chang et al, 2013 | Taiwan (Tainan City) | No | plasma | Yes | Glucose oxidase method | DSM-IV (MINI) |
| Chang et al, 2017 | Taiwan (Tainan City) | Lorazepam max. 6 mg | plasma | Yes | High-sensitivity CRP ELISA kit | DSM-IV (MINI) |
| Chiou et al, 2017 | Taiwan (Kaohsiung) | No | Serum | NA | ELISA | DSM-IV TR |
| Chiou et al, 2017 | Taiwan (Kaohsiung) | No | Serum | NA | ELISA | DSM-IV |
| De Witte et al, 2014 | Germany and the Netherlands | No | Serum | Not all | HumanMAP  immunoassay platform | DSM-IV |
| Ding et al, 2014 | China | No | Plasma | NA | ELISA | DSM IV |
| Goff et al, | China (Shanghai metropolitan area) | No | Plasma | Yes | ELISA | DSM IV |
| Gonzalez-Pinto et al, 2010 | Spain (Vitoria, Alava) | No | Plasma | NA | ELISA | DSM IV (SCID-I) |
| Haring et al, 2015 | Estonia (Tartu) | No | Serum | NA | High-sensitive biochip array technology | ICD-10 |
| Ho et al, | Taiwan (Beitou) | No | Serum | Yes | Multiplex bead array assays (Luminex) | DSM-IV |
| Huang et al, 2006 | Taiwan (Kaohsiung) | No | Serum | Yes | ELISA | DSM-IV |
| Jordan et al, 2018 | Germany (Magdeburg) | Benzodiazepines for ≤6 days | Serum (CRP); Plasma (BDNF) | Yes | Latex technology immunoassay (CRP); Human BDNF DuoSet ELISA kit (BDNF) |  |
| Jordan et al, 2018 | Germany (Magdeburg) | No | Serum (CRP) and plasma (BDNF) | Yes | Latex technology immunoassay (CRP) and ELISA (BDNF) |  |
| Kavzoglu and Hariri et al, 2013 | Turkey (Erenköy) | No | Whole blood sample | Yes | Standard enzymatic procedures | DSM IV (SCID-I) |
| Leo et al, 2006 | Italy (Rome) | No | Plasma | Yes | ELISA | DSM-IV-TR  (SCID) |
| Li et al, 2013 | China | No | Plasma | Yes | ELISA | DSM-IV |
| Lin et al, 2018 | China (Shanghai) | Benzodiazepines | Serum | Yes | Multiplex kits |  |
| Lu et al, 2004 | China | No | Serum | Yes | ELISA |  |
| Martocchia et al, 2014 | Italy | No | Serum | NA | ELISA | DSM-IV-TR (SCID-I) |
| Muthuramalingam et al, 2016 | India (Puducherry, rural area) | No | NA | NA | ELISA | DSM-IV-TR (MINI) |
| Narula et al, 2010 | India | No | Serum | Yes |  | ICD -10 |
| Noto et al, 2016 | Brazil (Sao Paulo) | No | Serum | NA | Flow cytometry using the Cytometric Bead Array | DSM-IV (SCID-I) |
| Palomino et al, 2006 | Vitoria (Alava, Spain) | No | Plasma | NA | ELISA | DSM IV (SCID-I) |
| Ristevska-Dimitrovska et al, 2013 [Macedonian study] | Macedonia | No | Serum | NA | ELISA | ICD-10 |
| Rizos et al, 2014 | Spain | Lorazepam | Serum | NA | ELISA | DSM-IV |
| Saddichha et al, 2008 | India (Ranchi) | Herbal treatments | Serum | Yes | NA | DSM-IV |
| Skibinska et al, 2018 | Poland (Poznań) | No | Serum | Yes | ELISA | DSM-IV  (SCID) |
| Song et al, 2009 | China | No | Serum | NA | ELISA | DSM-IV |
| Song et al, 2014 | China | No | Serum | NA | ELISA | DSM-IV |
| Stefanović et al, 2015 | Serbia (Belgrade) | No |  | Yes | Hematology analyzer | ICD-10 |
| Sutcigil et al, 2007 | Turkey (Ankara) | No | Serum | Yes | Enzyme immunoassay method kits (Bender MedSystems) | DSM-IV |
| Theodoropoulou et al, 2001 | Greece | No | Serum | NA | ELISA | DSM-III-R |
| Wani et al, 2015 | India (North India) | No | Plasma | Yes | Enzymatic photometric test | DSM-IV TR |
| Wu et al, 2018 | China (Shanghai) | NA | Serum | Yes | ELISA | CCMD‑3‑R |
| Yoshimura et al, 2012 | Japan | Lorazepam and biperiden | Serum | NA | ELISA | DSM-IV |
| Yoshimura et al, 2014 | Japan | Benzodiazepines | Serum | NA | ELISA | DSM-IV |
| Yuan et al, 2018 | China | No |  | Yes | Particle-enhanced immunoturbidimetric assay (CRP); standard enzymatic methods and an automated analyzer (fasting glucose) | DSM-IV |
| Zhai et al, 2017 | China | No | Serum | Yes |  | ICD-10 |
| Zhou et al, 2019 | China | No | Serum | Yes | AU680 Chemistry Analyzer | DSM-V |
| Abbreviations: BDNF, brain-derived neurotrophic factor; CCMD‑3‑R, Chinese Classification of Mental Disorders 3, Revised Third Edition; CRP, C-reactive protein; DSM, Diagnostic and Statistical Manual of Mental Disorders; ELISA, Enzyme-linked immunosorbent assay; ICD, International Statistical Classification of Diseases and Related Health Problems; Major Depressive Disorder; MINI, Mini International Neuropsychiatric Interview ; NA, not applicable; SCID-I, Structured Clinical Interview for DSM-IV; TNFα, tumor necrosis factor alpha. | | | | | | |
|  |  |  |  |  |  |  |
|  |  |  |  |  |  |  |

| **Table 6. In- and exclusion criteria from the included studies** | | |
| --- | --- | --- |
| **Study** | **Inclusion criteria** | **Exclusion criteria** |
| Aydemir et al, 2006 | Female patients who applied to the out-patient clinic and diagnosed as major depressive disorder. Patients' ages ranged between 17 and 45 years. | Other major axis I disorders, including schizophrenia, bipolar disorders, anxiety disorders, substance-related disorders and eating disorders, as well as the presence of any physical disorders and/or exposure to any drugs including antidepressants and oral contraceptives in the past 8 weeks. |
| Basoglu et al, 2010 | Patients were referred to an educational military health center owing to their psychotic behavior during their mandatory military service. Participants were all hospitalized first episode, drug naive patients. | Alcohol and substance abuse or dependence (other than smoking), having an important medical problem such as Wilson’s disease, Down’s syndrome, malnutrition, diabetes mellitus, chronic renal failure, cancer, liver cirrhosis and thyroid diseases, known endocrinological illnesses, BMI greater than 30, past history of psychosis and use of antipsychotic drugs, severe neurological disorders such as epilepsy or use of antiepileptic drugs. |
| Borovcanin et al, 2013 | First-episode psychosis patients. | Acute or chronic medical conditions, especially having current infections, allergies or autoimmune disorders, or current anti-inflammatory or antiviral medications. Neither the psychotic patients nor controls suffered from substance or alcohol abuse, nor comorbidity of other mental illnesses were diagnosed. |
| Cai et al, 2012 | DSM-IV-TR criteria for schizophrenia; first time hospitalized for psychiatric illness and never exposed to neuroleptics; aged 18−50 years; and from the same geographic region and of the same ethnic origin. Additionally, all the participants were non-smokers. | A history of substance abuse, chronic use of any other medication, CNS impairment, past or present neurological disease, or any other organic disorder. |
| Chang et al, 2013 | MDD outpatients. | Monoamine oxidase inhibitor or any class of antidepressant treatment prior to entering the study; a DSM-IV diagnosis of substance abuse within the past three months; anorganic mental disease, mental retardation or dementia; a serious surgical condition or physical illness; patients who were pregnant or breast feeding; patients who were previously diagnosed with type 2 diabetes mellitus ;and patients who have HbA1c > 46%. |
| Chang et al, 2017 | Drug-naïve MDD outpatients with a first diagnosis and HDRS scores > 15. | A serious surgical condition or physical illness (including all chronic diseases, heart disease, stroke, kidney dialysis, transplant, etc.); patients who were pregnant or breastfeeding; a DSM-IV diagnosis of substance abuse within the past 3 months; previous use of any psychotropic agent; taking any anti-inflammatory medication before, during the trial period, having autoimmune disorder, or at infectious status (CRP>7mg/l); and an organic mental disease, mental retardation or dementia. |
| Chiou et al, 2017 | Patients with first-episode drug-naïve schizophrenia. | Any alcohol abuse, drug abuse, pregnancy, physical illness or organic brain disorder. |
| Chiou et al, 2017 | First-episode drug-naïve MDD. | 17-item Hamilton Depression Rating Scale <20. Chronic medical illness including liver, kidney, lung, heart, and metabolic diseases. They had neither allergic reactions nor acute infections. |
| De Witte et al, 2014 | First onset and antipsychotic-naïve schizophrenia patients. | Serious medical conditions such as type II diabetes mellitus, hypertension, cardiovascular or autoimmune diseases. |
| Ding et al, 2014 | Patients with schizophrenia aged 18 to 45 years; disease duration less than 2 years; the Positive and Negative Syndrome Scale total score ≥60. | Prior antipsychotic treatment; previous history of alcohol or other substance use; additional psychiatric diagnosis other than schizophrenia; certain medical conditions including acute infection, heart disease, epilepsy, hepatic or renal diseases, diabetes, aplastic anaemia, asthma or autoimmune diseases; female patients who were pregnant or lactating; and patients who were overweight or obese BMI ≥24 kg/m2 |
| Goff et al, 2018 | Nonaffective first-episode psychosis; Mandarin-speaking Han  Chinese individuals; ages of 16 and 40 years; right-handed; completed  at least 9 years of school. | Any other Axis I disorder, substance abuse, suicidal ideation, unstable medical illness, and contraindications  to magnetic resonance imaging |
| Gonzalez-Pinto et al, 2010 | Patients with a first psychotic episode which was defined as the first time displaying positive psychotic symptoms of delusions or hallucinations (within a psychotic episode) of less than 6 months duration. | Patients with mental retardation, organic brain disorders or drug abuse as a primary diagnosis were excluded. |
| Haring et al, 2015 | Patients with first episode psychosis and aged between 18 and 45. The duration of their untreated psychosis had been less than 3 years and they have not received antipsychotic treatment before the first contact with medical services for psychosis. | Psychotic disorders owing to a general medical condition or substance induced psychosis. |
| Ho et al, 2015 | All participants were over 20 years of age, drug naïve, in good health and diagnosed with clinically significant depression. | History of another significant medical disorder (e.g., cardiovascular disease, diabetes, or rheumatic disease); substance-related disorders; female; depression occurred after age 40, tobacco use and alcohol consumption |
| Huang et al, 2006 | Schizophrenic outpatients or inpatients. | No participants had any systemic diseases, including heart, liver, and thyroid diseases. |
| Jordan et al, 2018 | Drug-naïve first episode inpatients with schizophrenia. | Immune diseases, immunomodulating treatment, cancer, chronic terminal disease, cardiovascular disorders, diabetes or severe trauma. Psychosis or depression resulting from other medical conditions and substance-induced psychosis. |
| Jordan et al, 2018 | Drug-naïve first episode inpatients with MDD. | Immune diseases, immunomodulating treatment, cancer, chronic terminal disease, cardiovascular disorders, diabetes or severe trauma. Psychosis or depression resulting from other medical conditions and substance-induced psychosis |
| Kavzoglu and Hariri et al, 2013 | First episode of schizophrenia | Pregnancy, any significant disease which was detected in the general internal medicine examination and reports of any former diagnosis or treatment for a chronic cardiovascular, endocrinologic, hematologic, neurological or renal condition, a history of alcohol or substance abuse reported by their families. |
| Leo et al, 2006 | First-episode and drug naïve MDD in- and outpatients. | Medical or neurological diseases; other psychiatric disorders; conventional risk factors for CAD incl. hypertension, hyperlipidaemia, obesity, diabetes, family history and smoking; use of steroids, NSAIDs or antibiotics during the 2 preceding weeks; contraceptive hormonal therapy |
| Li et al, 2013 | First episode and drug-naive (treatment with traditional Chinese medications and acupuncture were also excluded) Chinese Han population patients with ages from 18 to 60 years old and HRSD-17 ≥17. | Smoking, drinking, substance dependence, severe medical illness (such as cancer, diabetes), brain disease, pregnancy, infectious, auto-immunological, endocrinological, pulmonary, cardiac, and hematological diseases. |
| Lin et al, 2018 | Inpatients with schizophrenia, age from 18 to 65 years old, Han Chinese; untreated with any antipsychotic medications or other psychotropics except benzodiazepines within 6 months prior to the study screening, including those who were never medicated with any antipsychotic treatment. | Medical conditions; concurrent diagnosis of psychiatric disorders defined in ICD-10 other than schizophrenia; concurrent treatment with other antipsychotics other than benzodiazepines, or use of anti-diabetic or lipid-lowering agents or special diets to lower glucose or lipid levels, or use of immunosuppressive agents; diabetes, dyslipidaemia, or endocrine disease; ongoing infections or allergies, history of alcohol or other substance use, autoimmune disorders, pregnancy or breastfeeding, known medical conditions that might affect metabolism, and history of diabetes or lipid disorders. |
| Lu et al, 2004 (China) | First-episode paranoid schizophrenia and have not used any antipsychotic drugs; PANSS score ≥ 65; | Organic diseases such as severe heart, brain, kidney and immune diseases. Recent vaccination, pregnancy, breastfeeding women. |
| Martocchia et al, 2014 | Naïve elderly patients with MDD. | Diagnosis of dementia, substance abuse, treatment with psychotropic agents (in particular, antidepressants, cholinesterase inhibitors or memantine) or glucocorticoids and obesity. |
| Muthuramalingam et al, 2016 | Drug naïve MDD aged 18-65 years | Suspected organic mood disorders, clinical evidence of fever or active infection, comorbid conditions such as systemic lupus erythematosus, diabetes or hypothyroidism and those receiving immunomodulatory. They also excluded those who required antidepressants other than fluoxetine in order to maintain treatment homogeneity. |
| Narula et al, 2010 | First-episode drug-naïve  Patients with schizophrenia. | History of any other neuropsychiatric  illness; on Selective Serotonin Reuptake Inhibitors (SSRIs), mood stabilizers or any other drug which could potentially influence the weight; positive substance abuse diagnosis in last 3 months; or a significant medical disorder. Pregnant and lactating women and women of childbearing  age not using adequate contraception were excluded. |
| Noto et al, 2016 | Drug naïve first episode psychosis patients with acute symptoms. Only patients between 16 and 40 years of age,who had never used any antipsychotic drugs before were included. | Psychotic episodes due to a general medical condition, substance-induced psychotic disorder, or intellectual disability, lifetime history of major psychiatric disorder. Acute and chronic medical conditions associated with an imbalance in the immune system, including infections (e.g., HIV), allergic reactions, pregnancy, the postpartum period, rheumatic disorders, and using medications with immunomodulatory effects such as nonsteroidal anti-inflammatory drugs, corticosteroids, and immunosuppressants. |
| Palomino et al, 2006 | First psychotic episode of schizophrenia. | Mental retardation, organic brain disorders, or drug abuse as a primary diagnosis. |
| Ristevska-Dimitrovska et al, 2013 [Macedonian study] | First depressive episode. |  |
| Rizos et al, 2014 | Drug-naive patients in their first-episode of schizophrenia. | Deterioration in their clinical state, which would require changes in the antipsychotic  treatment, regarding the type of the antipsychotic agent. Substance abuse. |
| Saddichha et al, 2008 | Medication-naïve patients with schizophrenia. | Other psychiatric comorbidity, history of severe physical illness, alcohol and substance abuse or dependence and history of pre-existing diabetes or hypertension or family history of hypertension or DM. |
| Skibinska et al, 2018 | Patients with a first-episode of depression with a HDRS-17 baseline score ≥ 25 from a native Polish population. | Chronic or acute somatic or neurological diseases and increased CRP level. |
| Song et al, 2009 | First-episode schizophrenia aged 16-45 years who had not been treated with neuroleptics before recruitment. | Subjects with ongoing infections, allergies, or past history of alcohol or other substance abuse/dependence and autoimmune disorders were excluded. None of the schizophrenic patients or control subjects were taking immunosuppressive drugs. None of the patients had been treated with neuroleptics before recruitment. |
| Song et al, 2014 | Inpatients from 18-45 years old diagnosed with first-episode schizophrenia and who were never previously treated with antipsychotic medications or other psychotropics. | Ongoing infections or allergies, history of alcohol or other substance use, autoimmune disorders, pregnancy, known medical conditions that might affect metabolism, history of diabetes or lipid disorder, use of anti-diabetic or lipid-lowering agents, or special diets to lower glucose or lipid levels, use of immunosuppressive agents and BMI ≥24. |
| Stefanović et al, 2015 | Patients with first-episode schizophrenia, agend between 18 and 45 years, both genders, and who have not previously received antipsychotic drugs (drug naive). | Comorbidity with inflammatory, neurodegenerative, malignant diseases, congestive heart disease and infectious diseases, as well as patients who were identified as alcohol or psychoactive substance abusers. |
| Sutcigil et al, 2007 | First episode MDD outpatients. | Other Axis I or Axis II diagnosis; pregnancy; acute or chronic infections within the past month; autoimmune, allergic, neoplastic or endocrine diseases and other acute physical disorders within past 3 months; use of antidepressants, NSAIDs or oral contraceptives in the past 6 weeks |
| Theodoropoulou et al, 2001 | First-episode drug-naive schizophrenic patients | Clinical or laboratory signs of physical illness affecting their immunological state |
| Wani et al, 2015 | First episode and drug naive schizophrenia subjects. | Medical conditions that could confound glucoregulatory assessments including history of diabetes mellitus, cardiovascular, and respiratory conditions with hemodynamic compromise or hypoxia, malignancy, epilepsy, endocrine conditions (excluding corrected thyroid abnormalities), current fever, pregnancy or high dose estrogen therapy, narcotic, corticosteroid or spironolactone therapy, sedative hypnotic withdrawal, any change in medication within 10 days of study. |
| Wu et al, 2018 | First‑episode schizophrenia without any history of antipsychotic drug treatment. | Severe dementia and organic disease, intolerance to the serious side effects of the drug (to be received), and not receiving transcranial magnetic stimulation treatment. |
| Yoshimura et al, 2012 | First-episode untreated schizophrenia patients | Current or past serious medical or neurological illness, or dependence on alcohol or illicit substances. |
| Yoshimura et al, 2014 | Drug-naïve and first-episode patients with MDD. Physically healthy. | Current alcohol or drug abuse, comorbid  anxiety, or personality disorders. |
| Yuan et al, 2018 | Diagnosis of first episode schizophrenia and never been on antipsychotic  medication before; PANSS total score >60 points; born through normal vaginal delivery; normal body weight  (BMI: 18.5–23.0). | (1) diagnoses of autoimmune diseases, heart diseases, hepatobiliary and gastrointestinal diseases, blood diseases, diabetes neurological diseases, or psychiatric diseases other than first episode schizophrenia; (2) pregnant or lactating women; (3) a history of using any antibiotic or anti-inflammatory agent, or probiotic in the past month; (4) a significant  change in the living environment or diet in the past month; (5) significant diarrhea or constipation in the past month. |
| Zhai et al, 2017 | First-episode schizophrenia and never previously treated with antipsychotic medications. | Bipolar disorder, major depressive disorder with psychosis, substance induced psychotic disorder, or psychotic disorder due to a general medical condition; having clinically significant head trauma or another serious medical condition; ongoing pregnancy, known medical conditions that might affect metabolism, history of diabetes, use of anti-diabetic or lipid-lowering agents, or special diets to lower glucose or lipid levels; alterations of hepatic or renal function; history of alcohol or other substance dependence. |
| Zhou et al, 2019 | First psychotic episode of schizophrenia diagnosed and not previously been treated with antipsychotic drug treatment. | Any psychiatric disorder other than schizophrenia (according to DSM-V); history of any substance–related and addictive disorders met DSM-V criteria; a significant risk of committing suicide based on history or mental status exam; pregnant or lactating patients; specific systemic diseases; or other medical conditions such as diabetes mellitus, dyslipidaemia, cardiovascular diseases, and hypertension; clinically significant abnormal laboratory test results, vital sign, or electrocardiogram findings. |
| Abbreviations: CRP, C-reactive protein; DM, diabetes mellitus; FEP, First-Episode Psychosis; HDRS, Hamilton Depression Rating Scale; HAM-D, Hamilton Rating Scale for Depression; Major Depressive Disorder; PANSS, Positive and Negative Syndrome Scale. | | |
|  |  |  |
|  |  |  |

| **Table 7. Statistical Comparison of Effect Sizes in Schizophrenia versus MDD Using a Wald-Type Test** | | |
| --- | --- | --- |
| **Compound** | **z-score** | ***p*-value** |
| BDNF | 0.155 | 0.877 |
| CRP | 0.923 | 0.356 |
| IL-1β | 0.343 | 0.732 |
| IL-2 | 0.531 | 0.595 |
| IL-4 | -1.801 | 0.070 |
| IL-6 | -0.316 | 0.752 |
| TNFα | 1.585 | 0.113 |
| FG | 1.409 | 0.159 |
|  | | |
| **System** | **z-score** | ***p*-value** |
| Immune | 0.676 | 0.499 |
| Abbreviations: BDNF, Brain-Derived Neurotrophic Factor; CRP, C-reactive protein; FG, fasting glucose; IL, Interleukin; MDD, Major Depressive Disorder; TNFα, Tumor necrosis factor alpha. | | |

| **Table 8. Newcastle-Ottawa Quality Assessment Scale for Cohort Studies** | | | | |
| --- | --- | --- | --- | --- |
|  |  |  |  |  |
| **Source** | **Selection** | **Comparability** | **Exposure** | **Total** |
| Aydemir et al, 2006 | ******* | ****** | ***** | 6 |
| Başoğlu et al, 2010 | ******* | ***** | ***** | 4 |
| Borovcanin et al, 2013 | ******* |  | * | 4 |
| Cai et al, 2012 | ******* |  | ******* | 6 |
| Chang et al, 2013 | ****** | ****** | ***** | 5 |
| Chang et al, 2017 | ******* | ****** | ****** | 7 |
| Chiou et al, 2017 | ******* | ****** | ***** | 6 |
| Chiou et al, 2017 | ******* | ****** | ***** | 6 |
| De Witte et al, 2014 | ******* | ****** | ***** | 6 |
| Ding et al, 2014 | ******* | ****** | ****** | 7 |
| Goff et al, 2018 | ******* |  | ***** | 4 |
| Gonzalez-Pinto et al, 2010 | ****** |  | ***** | 3 |
| Haring et al, 2015 | ******* | ****** | ****** | 7 |
| Ho et al, 2015 | ******* | ***** | ****** | 6 |
| Huang et al, 2018 | ******* |  | ******* | 6 |
| Jordan et al, 2018 | ******* | ****** | ***** | 6 |
| Kavzoglu and Hariri et al, 2013 | ******* | ***** | ***** | 5 |
| Kuwano et al, 2018 | ****** |  | ****** | 4 |
| Leo et al, 2006 | ******* | ***** | ****** | 6 |
| Li et al, 2013 | ******* | ****** | ******* | 8 |
| Lin et al, 2018 | ******* | ****** | ***** | 6 |
| Lu et al, 2004 | ******* |  | ***** | 4 |
| Martocchia et al, 2014 | ****** |  | ***** | 3 |
| Muthuramalingam et al, 2016 | ******* |  | ***** | 4 |
| Narula et al, 2010 | ****** |  | ******* | 5 |
| Noto et al, 2016 | ******* | ****** | ***** | 4 |
| Palomino et al, 2006 | ****** | ****** | ***** | 5 |
| Ristevska-Dimitrovska et al, 2013 [Macedonian study] | ***** | ***** | ***** | 3 |
| Rizos et al, 2014 | ******* | ***** | ****** | 6 |
| Saddichha et al, 2008 | ******* |  | ****** | 5 |
| Skibinska et al, 2018 | ******** | ***** | ***** | 6 |
| Song et al, 2009 | ******* |  | ****** | 5 |
| Song et al, 2014 | ******* | ****** | ****** | 7 |
| Stefanović et al, 2015 | ******* |  | ***** | 4 |
| Sutcigil et al, 2007 | ******** |  | ****** | 6 |
| Theodoropoulou et al, 2001 | ****** |  | ***** | 3 |
| Wani et al, 2015 | ****** |  | ***** | 3 |
| Wu et al, 2018 | ******* |  | ****** | 5 |
| Yoshimura et al, 2012 | ******* |  | ***** | 4 |
| Yoshimura et al, 2014 | ****** |  | ***** | 3 |
| Yuan et al, 2018 | ******* | ****** | ****** | 6 |
| Zhai et al, 2017 | ****** |  |  | 2 |
| Zhou et al, 2019 | ******* | ***** | ****** | 6 |
| Quality points for selection were: origin defined of patient group = ***;** independent validation of disorder = *****; use of validated assay (description needed); no other important medical conditions (e.g. diabetes, hypertension, cardiovascular disease or autoimmune disease). Quality points for comparability were: patients matched for age or adjusted for age in analysis = *****; patients matched for sex or adjusted for sex in analysis. Quality points for outcome were: blind assessment = *****; follow-up period lasted for at least 6 weeks = *****; attrition rate ≤ 20%= *****. | | | | |
|  |  |  |  |  |
|  |  |  |  |  |

| **Table 9. Effect Sizes for Compounds Measured in High-Quality Studies Only** | | | | | | | |
| --- | --- | --- | --- | --- | --- | --- | --- |
|  |  |  |  |  |  |  |  |
|  | | | | | **95% CI** | |  |
| **Compounds** | **Disorder** | **Studies** | **Sample Size, No.** | **Hedges' *g*** | **Lower Limit** | **Upper Limit** | ***p*-value** |
| BDNF | Schizophrenia | 3 | 62 | 0.51 | 0.14 | 0.88 | **0.007** |
|  | MDD | 4 | 109 | 0.30 | -0.23 | 0.82 | 0.273 |
| CRP | Schizophrenia | 2 | 59 | 0.67 | -0.01 | 1.35 | **0.055** |
|  | MDD | 2 | 90 | -0.14 | -0.87 | 0.59 | 0.705 |
| IL-1β | Schizophrenia | 1 | 33 | -0.02 | -0.35 | 0.31 | 0.902 |
|  | MDD | 2 | 20 | -0.42 | -1.40 | 0.55 | 0.393 |
| IL-2 | Schizophrenia | 1 | 33 | -1.38 | -1.85 | -0.91 | **< 0.001** |
|  | MDD | 2 | 49 | -0.98 | -3.82 | 1.86 | 0.500 |
| IL-4 | Schizophrenia | 1 | 33 | -0.40 | -0.75 | -0.06 | 0.022 |
|  | MDD | 2 | 49 | 1.47 | -0.83 | 3.77 | 0.210 |
| IL-6 | Schizophrenia | 3 | 155 | -0.15 | -0.59 | 0.28 | 0.492 |
|  | MDD | 2 | 46 | -0.44 | -1.33 | 0.46 | 0.340 |
| TNFα | Schizophrenia | 2 | 61 | 0.14 | -0.78 | 1.05 | 0.768 |
|  | MDD | 4 | 130 | -1.14 | -2.25 | -0.03 | **0.045** |
| Glucose | Schizophrenia | 5 | 157 | 0.15 | -0.19 | 0.48 | 0.402 |
|  | MDD | 1 | 30 | -0.75 | -1.54 | 0.03 | 0.061 |
| Only high-quality studies were included for analysis, as assessed with the Newcastle-Ottawa quality assessment scale for cohort studies. As a cut-off value for high quality we chose ≥6. Abbreviations: BDNF, brain-derived neurotrophic factor, CRP, C-reactive protein; IL, interleukin; MDD, major depressive disorder; TNFα, tumor necrosis factor alpha. | | | | | | | |

| \| **Table 10. Effects of Psychopharmacological Treatment Types on Effect Sizes** \| \| --- \| \|  \| | | | | | | | |
| --- | --- | --- | --- | --- | --- | --- | --- | --- | --- |
| **Compounds** | **Disorder** | **Treatment** | **Analyses** | **Hedges' *g*** | **Lower Limit** | **Upper Limit** | ***p*-value** |
| BDNF | Schizophrenia | Atypical AP | 8 | 0.53 | 0.37 | 0.70 | **0.000** |
|  |  | Aripiprazole | 1 | 0.61 | 0.02 | 1.20 | **0.041** |
|  |  | Olanzapine | 1 | 0.45 | -0.10 | 1.01 | 0.111 |
|  |  | Paliperidone | 1 | 0.65 | 0.34 | 0.96 | **< 0.001** |
|  |  | Risperidone | 1 | 0.57 | 0.26 | 0.87 | **< 0.001** |
|  | MDD | SSRI | 4 | 0.77 | 0.29 | 1.25 | **0.002** |
|  |  | Escitalopram | 2 | 0.90 | 0.27 | 1.54 | **0.005** |
|  |  | Fluvoxamine | 1 | 0.16 | -0.39 | 0.72 | 0.564 |
|  |  | Venlafaxine | 1 | -0.22 | -0.57 | 0.13 | 0.221 |
| CRP | Schizophrenia | Atypical | 2 | 0.67 | -0.01 | 1.35 | 0.055 |
|  |  | Risperidone | 1 | 0.44 | 0.00 | 0.87 | **0.049** |
|  | MDD | SSRI | 1 | -0.39 | -0.63 | -0.15 | **0.001** |
|  |  | Fluoxetine | 1 | -0.39 | -0.63 | -0.15 | **0.001** |
| IL-1β | Schizophrenia | Atypical AP | 1 | -0.43 | -0.66 | -0.21 | **< 0.001** |
|  |  | Risperidone | 1 | -0.43 | -0.66 | -0.21 | **< 0.001** |
|  | MDD | SSRI | 3 | -0.42 | -1.40 | 0.55 | 0.393 |
|  |  | Citalopram | 1 | -0.77 | -1.42 | -0.11 | **0.023** |
|  |  | Escitalopram | 1 | 0.41 | 0.02 | 0.80 | **0.039** |
|  |  | Sertraline | 1 | -1.02 | -1.75 | -0.30 | **0.005** |
| IL-2 | Schizophrenia | Atypical | 2 | 0.30 | -0.05 | 0.64 | 0.091 |
|  |  | Clozapine | 1 | 0.35 | -0.18 | 0.88 | 0.192 |
|  |  | Risperidone | 1 | 0.25 | -0.19 | 0.70 | 0.267 |
|  | MDD | SSRI | 2 | -0.98 | -3.82 | 1.86 | 0.500 |
|  |  | Escitalopram | 1 | 0.45 | 0.06 | 0.84 | **0.025** |
|  |  | Sertraline | 1 | -2.45 | -3.26 | -1.64 | **< 0.001** |
| IL-4 | Schizophrenia | Atypical AP | 1 | -0.21 | -0.44 | 0.02 | 0.075 |
|  |  | Risperidone | 1 | -0.21 | -0.44 | 0.02 | 0.075 |
|  | MDD | SSRI | 2 | 1.47 | -0.83 | 3.77 | 0.210 |
|  |  | Escitalopram | 1 | 0.33 | -0.05 | 0.71 | 0.091 |
|  |  | Sertraline | 1 | 2.68 | 1.81 | 3.55 | **< 0.001** |
| IL-6 | Schizophrenia | Atypical AP | 5 | -0.31 | -0.67 | 0.04 | 0.083 |
|  |  | Clozapine | 1 | -0.65 | -1.21 | -0.08 | **0.025** |
|  |  | Risperidone | 4 | -0.25 | -0.64 | 0.14 | 0.203 |
|  | MDD | SSRI | 5 | -0.39 | -0.86 | 0.09 | 0.115 |
|  |  | Citalopram | 1 | -0.83 | -1.50 | -0.16 | **0.016** |
|  |  | Escitalopram | 1 | 0.32 | -0.06 | 0.71 | 0.096 |
|  |  | Fluoxetine | 2 | -0.39 | -0.75 | -0.03 | **0.034** |
|  |  | Sertraline | 1 | -0.91 | -1.61 | -0.22 | **0.010** |
| TNFα | Schizophrenia | Atypical AP | 4 | -0.41 | -0.99 | 0.17 | 0.170 |
|  |  | Clozapine | 1 | -0.77 | -1.36 | -0.18 | **0.010** |
|  |  | Olanzapine | 1 | 1.52 | -0.70 | 3.74 | 0.181 |
|  |  | Risperidone | 3 | -0.58 | -1.03 | -0.12 | **0.013** |
|  |  | Quetiapine | 1 | 1.83 | -0.61 | 4.27 | 0.141 |
|  | MDD | SSRI | 7 | -1.02 | -1.79 | -0.25 | **0.009** |
|  |  | Citalopram | 1 | -0.87 | -1.56 | -0.19 | **0.012** |
|  |  | Escitalopram | 1 | 0.45 | 0.05 | 0.84 | **0.026** |
|  |  | Fluoxetine | 2 | -0.75 | -1.78 | 0.27 | 0.149 |
|  |  | Sertraline | 2 | -1.35 | -1.97 | -0.73 | **< 0.001** |
|  |  | Venlafaxine | 1 | -2.77 | -3.64 | -1.89 | **< 0.001** |
| Glucose | Schizophrenia | Atypical AP | 14 | 0.36 | 0.08 | 0.63 | **0.011** |
|  |  | Typical AP | 2 | 0.70 | 0.18 | 1.23 | **0.009** |
|  |  | Aripiprazole | 1 | 0.68 | 0.11 | 1.26 | 0.019 |
|  |  | Haloperidol | 2 | 0.70 | 0.18 | 1.23 | **0.009** |
|  |  | Olanzapine | 6 | 0.50 | -0.02 | 1.03 | 0.061 |
|  |  | Paliperidone | 1 | 0.11 | -0.26 | 0.49 | 0.552 |
|  |  | Risperidone | 3 | 0.53 | 0.20 | 0.86 | **0.002** |
|  | MDD | SSRI | 2 | -0.26 | -1.04 | 0.51 | 0.510 |
| Subgroup analyses were performed to differentiate between the effects of the types of psychopharmacological treatment on blood compounds. Abbreviations: AP, antipsychotics; BDNF, brain-derived neurotrophic factor, CRP, C-reactive protein; IL, interleukin; MDD, major depressive disorder; n/a, no data available or there was no distinguish possible between treatment types; TNFα, tumor necrosis factor alpha. | | | | | | | |

**Supplementary Figure 2. Forest Plot Showing Effect Sizes of the Change of CRP following Treatment in Drug-Naïve First-Episode Schizophrenia and MDD**

**Supplementary Figure 3. Forest Plot Showing Effect Sizes of the Change of IL-1β following Treatment in Drug-Naïve First-Episode Schizophrenia and MDD**

**Supplementary Figure 4. Forest Plot Showing Effect Sizes of the Change of IL-2 following Treatment in Drug-Naïve First-Episode Schizophrenia and MDD**

**Figure 5. Forest Plot Showing Effect Sizes of the Change of IL-4 following Treatment in Drug-Naïve First-Episode Schizophrenia and MDD**

 **Figure 6A. Forest Plot Showing Effect Sizes of the Change of IL-6 following Treatment in Drug-Naïve First-Episode Schizophrenia and MDD**

**Figure 6B. Forest Plot Showing Effect Sizes of the Change of IL-6 following Treatment in Drug-Naïve First-Episode Schizophrenia and MDD (including two psychotherapy studies)**

**Figure 7. Forest Plot Showing Effect Sizes of the Change of Fasting Glucose following Treatment in Drug-Naïve First-Episode Schizophrenia and MDD**

**Figure 8.**

**
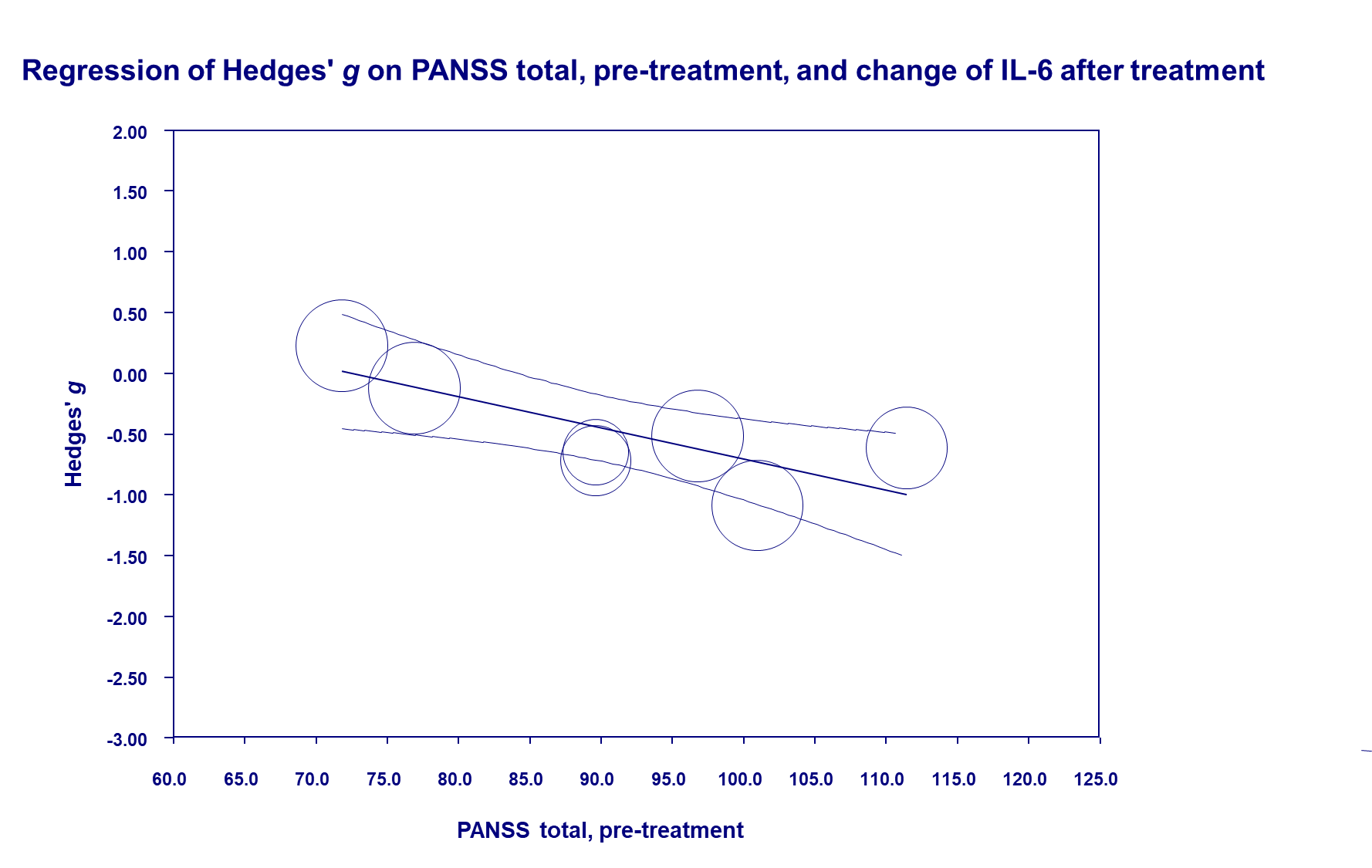
**

**Figure 9.**

**
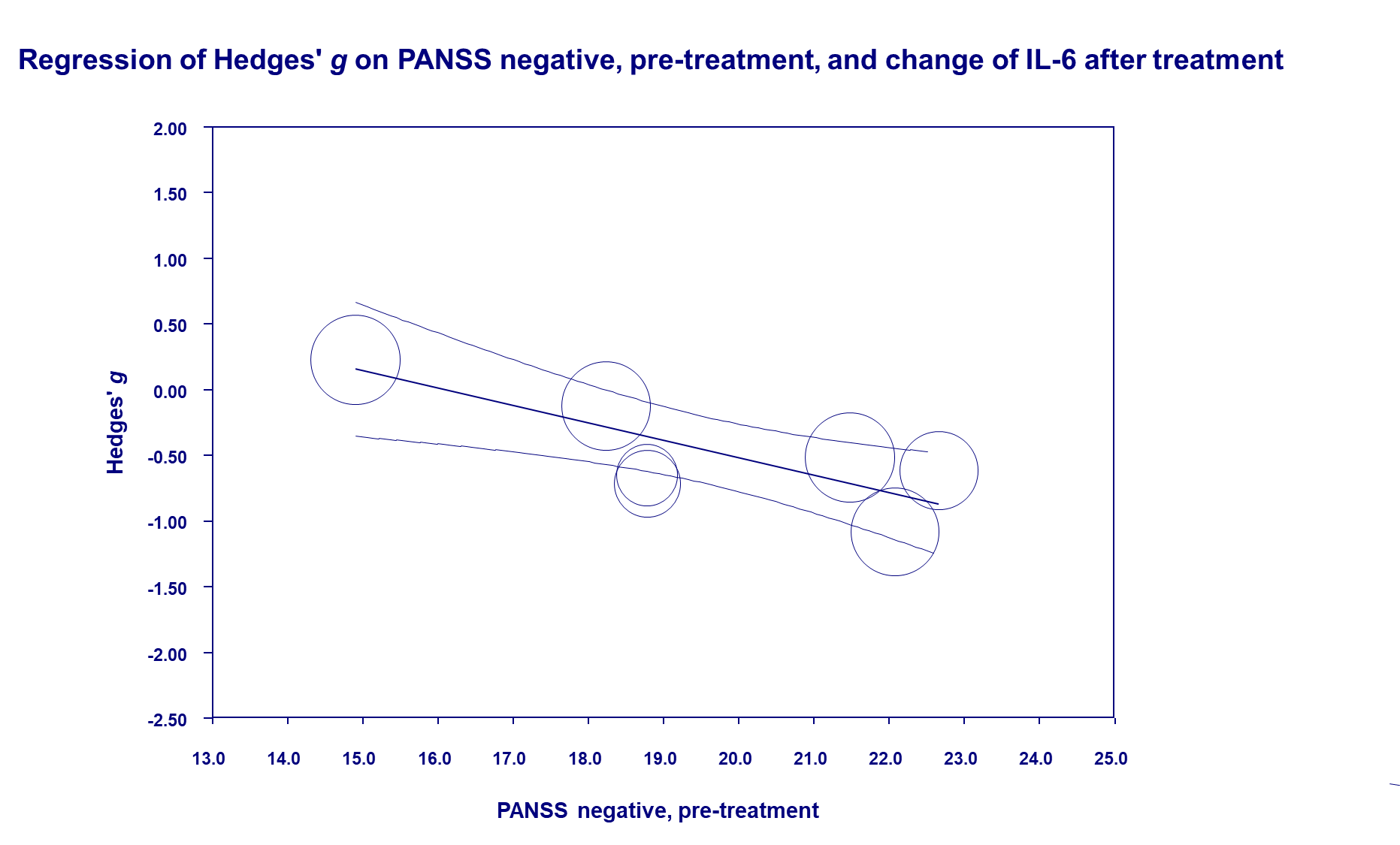
**

**Figure 10.**

**
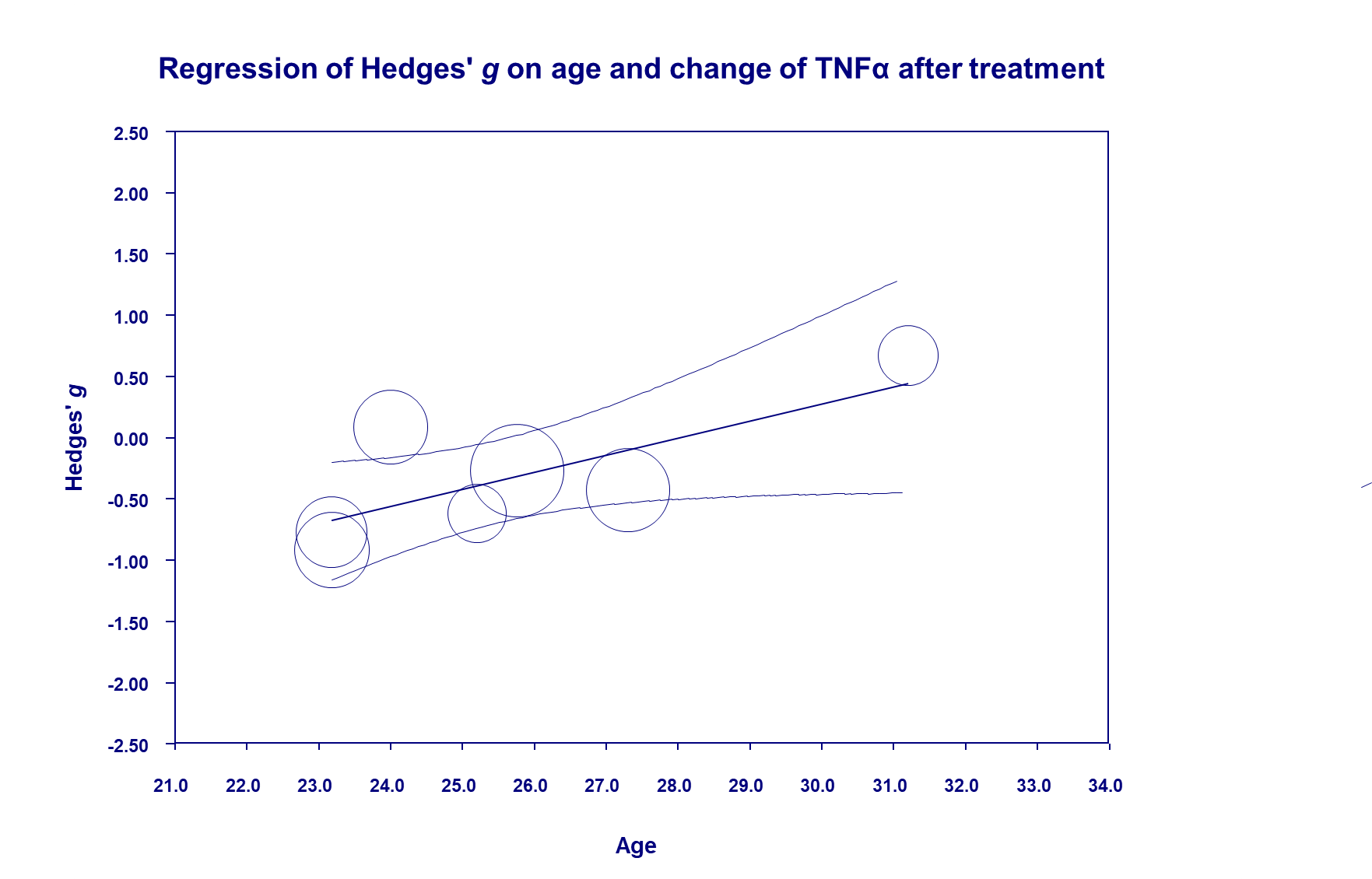
**

**Figure 11.**

**
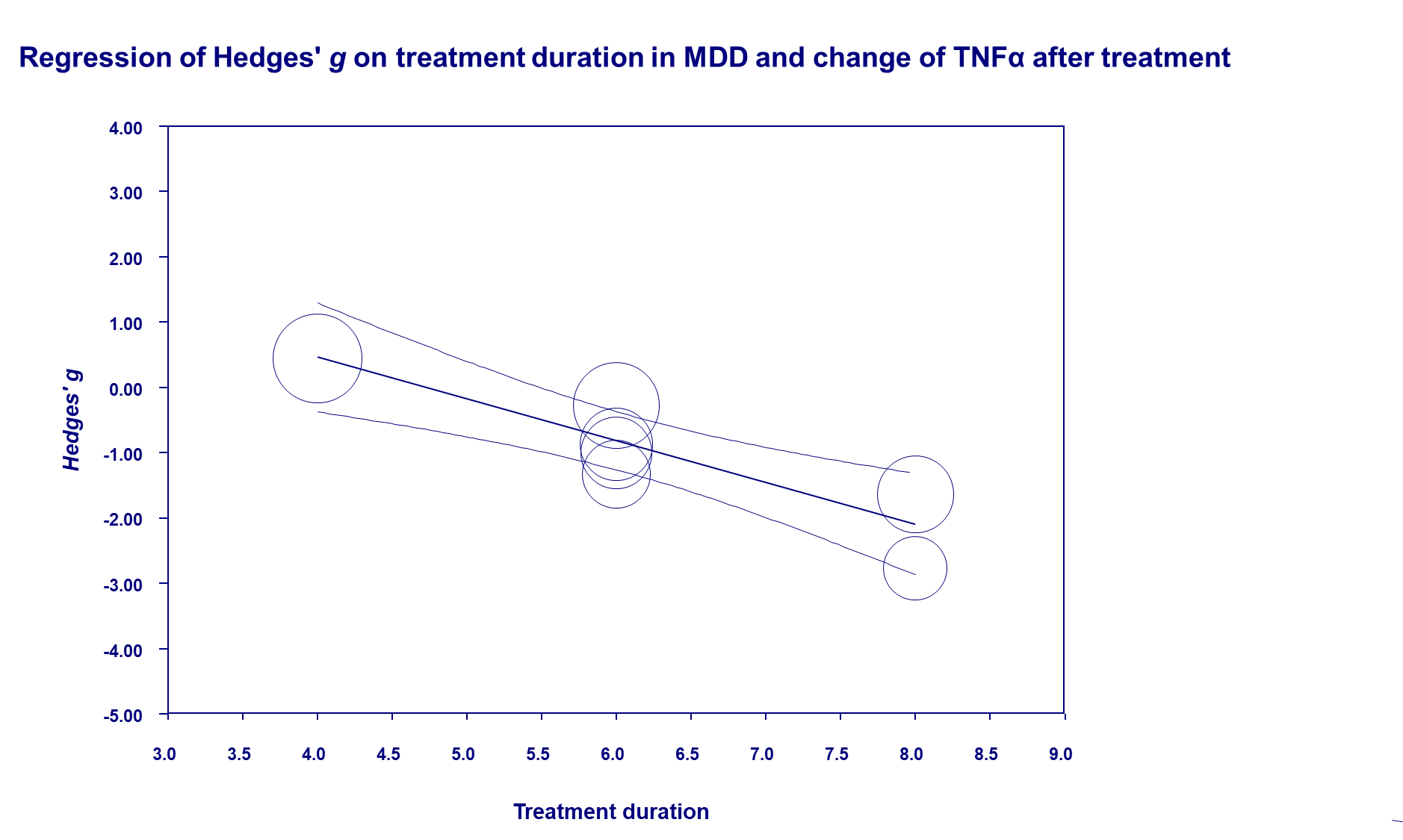
**

**Figure 12.**

**
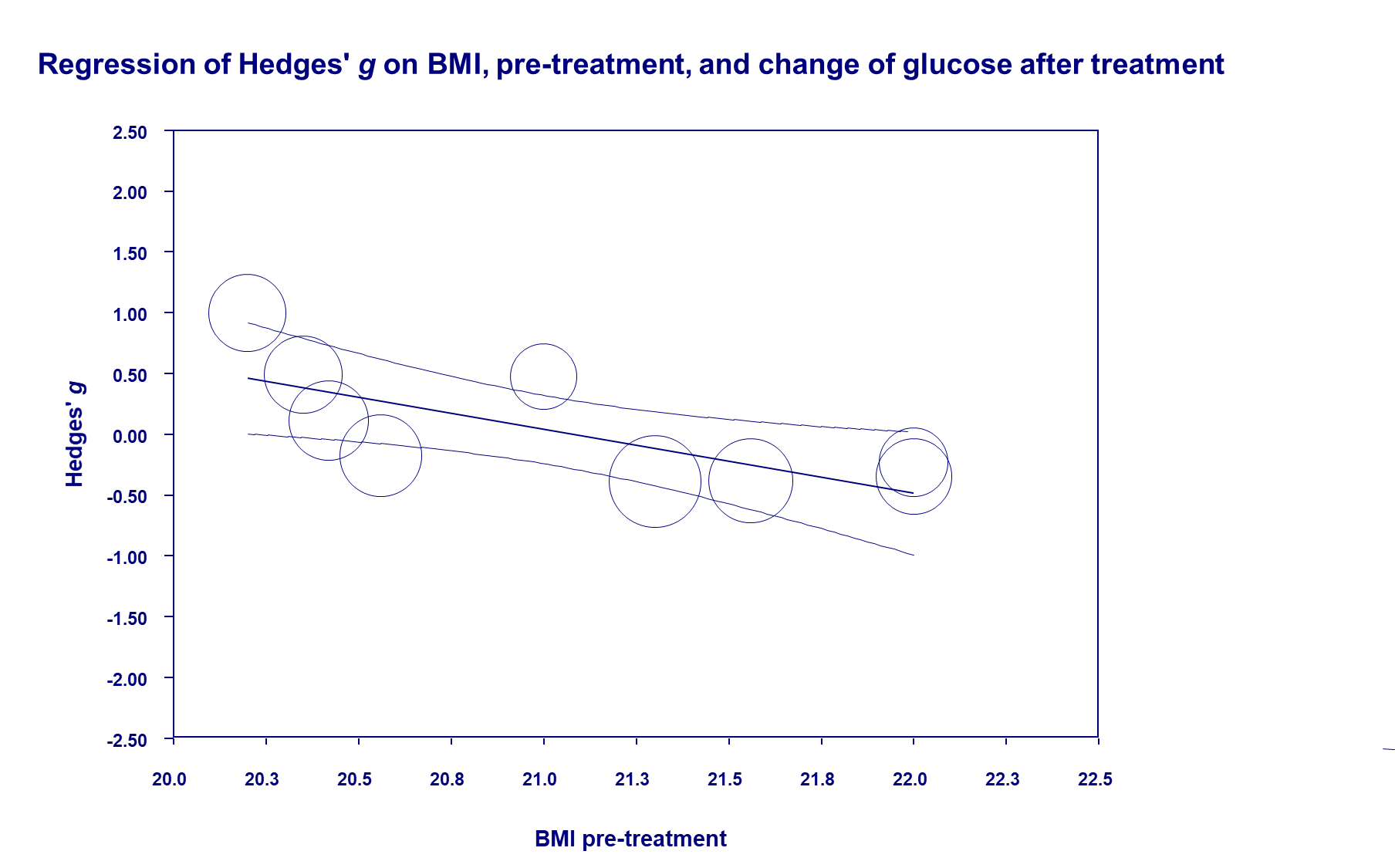
**

**
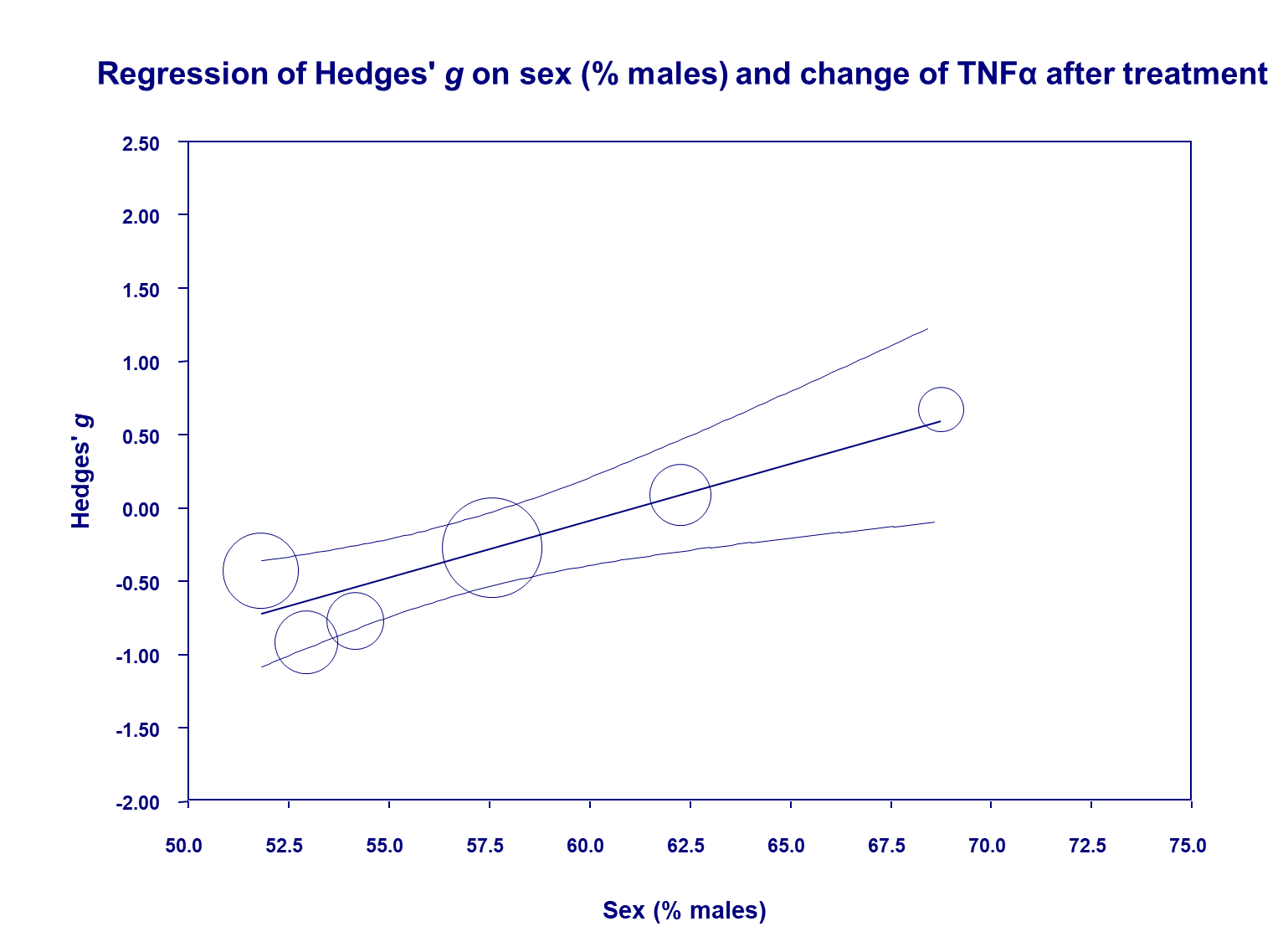
Figure 13**

**References**

Aydemir, C., Yalcin, E. S., Aksaray, S., Kisa, C., Yildirim, S. G., Uzbay, T., & Goka, E. (2006). Brain-derived neurotrophic factor (BDNF) changes in the serum of depressed women. *Progress in Neuro-Psychopharmacology and Biological Psychiatry*, *30*(7), 1256-60. doi:10.1016/j.pnpbp.2006.03.025.

Basoglu, C., Oner, O., Gunes, C., Semiz, U. B., Ates, A. M., Algul, A., . . . Ipcioglu, O. (2010). Plasma orexin A, ghrelin, cholecystokinin, visfatin, leptin and agouti-related protein levels during 6-week olanzapine treatment in first-episode male patients with psychosis. *International Clinical Psychopharmacology*, *25*(3), 165-71. doi:10.1097/YIC.0b013e3283377850.

Borovcanin, M., Jovanovic, I., Radosavljevic, G., Djukic Dejanovic, S., Stefanovic, V., Arsenijevic, N., & Lukic, M. L. (2013). Antipsychotics can modulate the cytokine profile in schizophrenia: attenuation of the type-2 inflammatory response. *Schizophrenia Research*, *147*(1), 103-109. doi:10.1016/j.schres.2013.03.027.

Cai, H. L., Li, H. D., Yan, X. Z., Sun, B., Zhang, Q., Yan, M., . . . Ye, H. S. (2012). Metabolomic analysis of biochemical changes in the plasma and urine of first-episode neuroleptic-naive schizophrenia patients after treatment with risperidone. *Journal of Proteome Research*, *11*(8), 4338-50. doi:10.1021/pr300459d.

Chang, H. H., Chi, M. H., Lee, I. H., Tsai, H. C., Gean, P. W., Yang, Y. K., . . . Chen, P. S. (2013). The change of insulin levels after six weeks antidepressant use in drug-naive major depressive patients. *Journal of Affective Disorders*, *150*(2), 295-9. doi:10.1016/j.jad.2013.04.008.

Chang, H. H., Wang, T. Y., Lee, I. H., Lee, S. Y., Chen, K. C., Huang, S. Y., . . . Chen, P. S. (2017). C-reactive protein: A differential biomarker for major depressive disorder and bipolar II disorder. *The World Journal of Biological Psychiatry*, *18*(1), 63-70. doi:10.3109/15622975.2016.1155746.

Chiou, Y. J., & Huang, T. L. (2017a). Serum Brain-Derived Neurotrophic Factors in Taiwanese Patients with Drug-Naive First-Episode Major Depressive Disorder: Effects of Antidepressants. *The International Journal of Neuropsychopharmacology*, *20*(3), 213-218. doi:10.1093/ijnp/pyw096.

Chiou, Y. J., & Huang, T. L. (2017b). Serum brain-derived neurotrophic factors in Taiwanese patients with drug-naive first-episode schizophrenia: Effects of antipsychotics. *The World Journal of Biological Psychiatry*, *18*(5), 382-391. doi:10.1080/15622975.2016.1224925.

de Witte, L., Tomasik, J., Schwarz, E., Guest, P. C., Rahmoune, H., Kahn, R. S., & Bahn, S. (2014). Cytokine alterations in first-episode schizophrenia patients before and after antipsychotic treatment. *Schizophrenia Research*, *154*(1-3), 23-9. doi:10.1016/j.schres.2014.02.005.

Ding, M., Song, X., Zhao, J., Gao, J., Li, X., Yang, G., . . . Lv, L. (2014). Activation of Th17 cells in drug naive, first episode schizophrenia. *Progress in Neuro-Psychopharmacology & Biological Psychiatry*, *51*(78-82. doi:10.1016/j.pnpbp.2014.01.001.

Fernandes, B. S., Steiner, J., Berk, M., Molendijk, M. L., Gonzalez-Pinto, A., Turck, C. W., . . . Goncalves, C. A. (2015). Peripheral brain-derived neurotrophic factor in schizophrenia and the role of antipsychotics: meta-analysis and implications. *Molecular Psychiatry*, *20*(9), 1108-19. doi:10.1038/mp.2014.117.

Goff, D. C., Zeng, B., Ardekani, B. A., Diminich, E. D., Tang, Y., Fan, X., . . . Wang, J. (2018). Association of Hippocampal Atrophy With Duration of Untreated Psychosis and Molecular Biomarkers During Initial Antipsychotic Treatment of First-Episode Psychosis. *JAMA Psychiatry*, *75*(4), 370-378. doi:10.1001/jamapsychiatry.2017.4595.

Gonzalez-Pinto, A., Mosquera, F., Palomino, A., Alberich, S., Gutierrez, A., Haidar, K., . . . Matute, C. (2010). Increase in brain-derived neurotrophic factor in first episode psychotic patients after treatment with atypical antipsychotics. *International Clinical Psychopharmacology*, *25*(4), 241-5.

Haring, L., Koido, K., Vasar, V., Leping, V., Zilmer, K., Zilmer, M., & Vasar, E. (2015). Antipsychotic treatment reduces psychotic symptoms and markers of low-grade inflammation in first episode psychosis patients, but increases their body mass index. *Schizophrenia Research*, *169*(1-3), 22-29. doi:10.1016/j.schres.2015.08.027.

Ho, P. S., Yeh, Y. W., Huang, S. Y., & Liang, C. S. (2015). A shift toward T helper 2 responses and an increase in modulators of innate immunity in depressed patients treated with escitalopram. *Psychoneuroendocrinology*, *53*(246-55. doi:10.1016/j.psyneuen.2015.01.008.

Huang, M., Yu, L., Pan, F., Lu, S., Hu, S., Hu, J., . . . Xu, Y. (2018). A randomized, 13-week study assessing the efficacy and metabolic effects of paliperidone palmitate injection and olanzapine in first-episode schizophrenia patients. *Progress in Neuro-Psychopharmacology & Biological Psychiatry,* *81*(122-130.

Jordan, W., Dobrowolny, H., Bahn, S., Bernstein, H. G., Brigadski, T., Frodl, T., . . . Steiner, J. (2018). Oxidative stress in drug-naive first episode patients with schizophrenia and major depression: effects of disease acuity and potential confounders. *European Archives of Psychiatry and Clinical Neuroscience*, *268*(2), 129-143. doi:10.1007/s00406-016-0749-7.

Kavzoglu, S. O., & Hariri, A. G. (2013). Intracellular Adhesion Molecule (ICAM-1), Vascular Cell Adhesion Molecule (VCAM-1) and E-Selectin Levels in First Episode Schizophrenic Patients. *Psychopharmacology*, 23(3), 205-214. doi:10.5455/bcp.20130713091100.

Kohler, C. A., Freitas, T. H., Maes, M., de Andrade, N. Q., Liu, C. S., Fernandes, B. S., . . . Carvalho, A. F. (2017). Peripheral cytokine and chemokine alterations in depression: a meta-analysis of 82 studies. *Acta Psychiatrica Scandinavica*, *135*(5), 373-387. doi:10.1111/acps.12698.

Kuwano, N., Kato, T. A., Setoyama, D., Sato-Kasai, M., Shimokawa, N., Hayakawa, K., . . . Kanba, S. (2018). Tryptophan-kynurenine and lipid related metabolites as blood biomarkers for first-episode drug-naive patients with major depressive disorder: An exploratory pilot case-control study. *Journal of Affective Disorders*, *231*, 74-82. doi:10.1016/j.jad.2018.01.014.

Leo, R., Di Lorenzo, G., Tesauro, M., Razzini, C., Forleo, G. B., Chiricolo, G., . . . Romeo, F. (2006). Association between enhanced soluble CD40 ligand and proinflammatory and prothrombotic states in major depressive disorder: pilot observations on the effects of selective serotonin reuptake inhibitor therapy. *The Journal of Clinical Psychiatry*, *67*(11), 1760-6.

Li, Z., Qi, D., Chen, J., Zhang, C., Yi, Z., Yuan, C., . . . Fang, Y. (2013). Venlafaxine inhibits the upregulation of plasma tumor necrosis factor-alpha (TNF-alpha) in the Chinese patients with major depressive disorder: a prospective longitudinal study. *Psychoneuroendocrinology*, *38*(1), 107-14. doi:10.1016/j.psyneuen.2012.05.005.

Lin, Y., Peng, Y., He, S., Xu, J., Shi, Y., Su, Y., . . . Cui, D. (2018). Serum IL-1ra, a novel biomarker predicting olanzapine-induced hypercholesterolemia and hyperleptinemia in schizophrenia. *Progress in Neuropsychopharmacology & Biological Psychiatry*, *84*, 71-78.

Lu, L. X., Guo, S. Q., Chen, W., Li, Q., Cheng, J., & Guo, J. H. (2004). Effect of clozapine and risperidone on serum cytokine levels in patients with first-episode paranoid schizophrenia [Chinese article]. *Di 1 Yi Jun Yi Da Xue Xue Bao = Academic Journal of the First Medical College of PLA*, *24*(11), 1251-4.

Martocchia, A., Curto, M., Scaccianoce, S., Comite, F., Xenos, D., Nasca, C., . . . Falaschi, P. (2014). Effects of escitalopram on serum BDNF levels in elderly patients with depression: a preliminary report. *Aging Clinical and Experimental Research*, *26*(4), 461-4. doi:10.1007/s40520-014-0194-2.

Molendijk, M. L., Spinhoven, P., Polak, M., Bus, B. A., Penninx, B. W., & Elzinga, B. M. (2014). Serum BDNF concentrations as peripheral manifestations of depression: evidence from a systematic review and meta-analyses on 179 associations (N=9484). *Molecular Psychiatry*, *19*(7), 791-800. doi:10.1038/mp.2013.105.

Muthuramalingam, A., Menon, V., Rajkumar, R. P., & Negi, V. S. (2016). Effect of Fluoxetine on Inflammatory Cytokines in Drug-Naive Major Depression: A Short-Term Prospective Study from South India. *Journal of Clinical Psychopharmacology*, *36*(6), 726-728. doi:10.1097/jcp.0000000000000606.

Narula, P. K., Rehan, H. S., Unni, K. E., & Gupta, N. (2010). Topiramate for prevention of olanzapine associated weight gain and metabolic dysfunction in schizophrenia: a double-blind, placebo-controlled trial. *Schizophrenia Research*, *118*(1-3), 218-23. doi:10.1016/j.schres.2010.02.001.

Noto, C., Ota, V. K., Santoro, M. L., Gouvea, E. S., Silva, P. N., Spindola, L. M., . . . Maes, M. (2016). Depression, Cytokine, and Cytokine by Treatment Interactions Modulate Gene Expression in Antipsychotic Naive First Episode Psychosis. *Molecular Neurobiology*, *53*(8), 5701-9. doi:10.1007/s12035-015-9489-3.

Palomino, A., Vallejo-Illarramendi, A., Gonzalez-Pinto, A., Aldama, A., Gonzalez-Gomez, C., Mosquera, F., . . . Matute, C. (2006). Decreased levels of plasma BDNF in first-episode schizophrenia and bipolar disorder patients. *Schizophrenia Research*, *86*(1-3), 321-2. doi:10.1016/j.schres.2006.05.028.

Pillinger, T., Beck, K., Gobjila, C., Donocik, J. G., Jauhar, S., & Howes, O. D. (2017). Impaired Glucose Homeostasis in First-Episode Schizophrenia: A Systematic Review and Meta-analysis. *JAMA Psychiatry*, *74*(3), 261-269. doi:10.1001/jamapsychiatry.2016.3803.

Rao, S., Martinez-Cengotitabengoa, M., Yao, Y., Guo, Z., Xu, Q., Li, S., . . . Zhang, F. (2017). Peripheral blood nerve growth factor levels in major psychiatric disorders. *Journal of Psychiatric Research*, *86*(39-45. doi:10.1016/j.jpsychires.2016.11.012.

Ristevska-Dimitrovska, G., Shishkov, R., Gerazova, V. P., Vujovik, V., Stefanovski, B., Novotni, A., . . . Filov, I. (2013). Different serum BDNF levels in depression: results from BDNF studies in FYR Macedonia and Bulgaria. *Psychiatria Danubina*, *25*(2), 123-7.

Rizos, E., Papathanasiou, M. A., Michalopoulou, P. G., Laskos, E., Mazioti, A., Kastania, A., . . . Liappas, I. (2014). A longitudinal study of alterations of hippocampal volumes and serum BDNF levels in association to atypical antipsychotics in a sample of first-episode patients with schizophrenia. *PLoS One*, *9*(2), e87997. doi:10.1371/journal.pone.0087997.

Saddichha, S., Manjunatha, N., Ameen, S., & Akhtar, S. (2008). Diabetes and schizophrenia - effect of disease or drug? Results from a randomized, double-blind, controlled prospective study in first-episode schizophrenia. *Acta Psychiatrica Scandinavica*, *117*(5), 342-7. doi:10.1111/j.1600-0447.2008.01158.x.

Skibinska, M., Kapelski, P., Rajewska-Rager, A., Pawlak, J., Szczepankiewicz, A., Narozna, B., . . . Dmitrzak-Weglarz, M. (2018). Brain-derived neurotrophic factor (BDNF) serum level in women with first-episode depression, correlation with clinical and metabolic parameters. *Nordic Journal of* Psychiatry, 72(3), 191-196.

Song, X., Fan, X., Li, X., Zhang, W., Gao, J., Zhao, J., . . . Lv, L. (2014). Changes in pro-inflammatory cytokines and body weight during 6-month risperidone treatment in drug naive, first-episode schizophrenia. *Psychopharmacology*, *231*(2), 319-25. doi:10.1007/s00213-013-3382-4.

Song, X. Q., Lv, L. X., Li, W. Q., Hao, Y. H., & Zhao, J. P. (2009). The interaction of nuclear factor-kappa B and cytokines is associated with schizophrenia. *Biological Psychiatry*, *65*(6), 481-8. doi:10.1016/j.biopsych.2008.10.018.

Stefanovic, V., Mihajlovic, G., Nenadovic, M., Dejanovic, S. D., Borovcanin, M., & Trajkovic, G. (2015). The effect of antipsychotic drugs on nonspecific inflammation markers in the first episode of schizophrenia. *Vojnosanitetski Pregled*, *72*(12), 1085-92. doi:10.2298/vsp140526016s.

Sutcigil, L., Oktenli, C., Musabak, U., Bozkurt, A., Cansever, A., Uzun, O., . . . Sengul, A. (2007). Pro- and anti-inflammatory cytokine balance in major depression: effect of sertraline therapy. *Clinical & Developmental Immunology*, *2007*(76396. doi:10.1155/2007/76396.

Theodoropoulou, S., Spanakos, G., Baxevanis, C. N., Economou, M., Gritzapis, A. D., Papamichail, M. P., & Stefanis, C. N. (2001). Cytokine serum levels, autologous mixed lymphocyte reaction and surface marker analysis in never medicated and chronically medicated schizophrenic patients. *Schizophrenia Research*, *47*(1), 13-25.

Upthegrove, R., Manzanares-Teson, N., & Barnes, N. M. (2014). Cytokine function in medication-naive first episode psychosis: a systematic review and meta-analysis. *Schizophrenia Research*, *155*(1-3), 101-8. doi:10.1016/j.schres.2014.03.005.

Wani, R. A., Dar, M. A., Margoob, M. A., Rather, Y. H., Haq, I., & Shah, M. S. (2015). Diabetes mellitus and impaired glucose tolerance in patients with schizophrenia, before and after antipsychotic treatment. *Journal of Neurosciences in Rural Practice*, *6*(1), 17-22. doi:10.4103/0976-3147.143182.

Wu, R. Q., Lin, C. G., Zhang, W., Lin, X. D., Chen, X. S., Chen, C., . . . Zhang, M. D. (2018). Effects of Risperidone and Paliperidone on Brain-Derived Neurotrophic Factor and N400 in First-Episode Schizophrenia. *Chinese Medical Journal (English)*, *131*(19), 2297-2301. doi:10.4103/0366-6999.241802.

Yoshimura, R., Hori, H., Ikenouchi-Sugita, A., Umene-Nakano, W., Katsuki, A., Hayashi, K., . . . Nakamura, J. (2012). Aripiprazole altered plasma levels of brain-derived neurotrophic factor and catecholamine metabolites in first-episode untreated Japanese schizophrenia patients. *Human Psychopharmacology*, *27*(1), 33-8. doi:10.1002/hup.1257.

Yoshimura, R., Kishi, T., Hori, H., Atake, K., Katsuki, A., Nakano-Umene, W., . . . Nakamura, J. (2014). Serum proBDNF/BDNF and response to fluvoxamine in drug-naive first-episode major depressive disorder patients. *Annals of General Psychiatry*, *13*(19. doi:10.1186/1744-859x-13-19.

Yuan, X., Zhang, P., Wang, Y., Liu, Y., Li, X., Kumar, B. U., . . . Song, X. (2018). Changes in metabolism and microbiota after 24-week risperidone treatment in drug naive, normal weight patients with first episode schizophrenia. *Schizophrenia Research*. doi:10.1016/j.schres.2018.05.017.

Zhai, D., Cui, T., Xu, Y., Feng, Y., Wang, X., Yang, Y., . . . Zhang, R. (2017). Cardiometabolic risk in first-episode schizophrenia (FES) patients with the earliest stages of both illness and antipsychotic treatment. *Schizophrenia Research*, *179*(41-49. doi:10.1016/j.schres.2016.09.001.

Zhou, X., Hu, M., Gong, M., Zou, X., & Yu, Z. (2019). Sex-differential effects of olanzapine vs. aripiprazole on glucose and lipid metabolism in first-episode schizophrenia. *Archives of Clinical Psychiatry (São Paulo)*, *46*(2), 33-9.
